# Metabolic Cooperation and Competition in the Tumor Microenvironment: Implications for Therapy

**DOI:** 10.3389/fonc.2017.00068

**Published:** 2017-04-12

**Authors:** Seema Gupta, Amrita Roy, Bilikere S. Dwarakanath

**Affiliations:** ^1^Georgia Cancer Center, Augusta University, Augusta, GA, USA; ^2^School of Life Sciences, B. S. Abdur Rahman Crescent University, Chennai, India; ^3^Central Research Facility, Sri Ramachandra University, Chennai, India

**Keywords:** tumor microenvironment, metabolic reprogramming, metabolic cooperation, Warburg effect, cancer-associated fibroblasts, immune network, cancer cell metabolism

## Abstract

The tumor microenvironment (TME) is an ensemble of non-tumor cells comprising fibroblasts, cells of the immune system, and endothelial cells, besides various soluble secretory factors from all cellular components (including tumor cells). The TME forms a pro-tumorigenic cocoon around the tumor cells where reprogramming of the metabolism occurs in tumor and non-tumor cells that underlies the nature of interactions as well as competitions ensuring steady supply of nutrients and anapleoretic molecules for the tumor cells that fuels its growth even under hypoxic conditions. This metabolic reprogramming also plays a significant role in suppressing the immune attack on the tumor cells and in resistance to therapies. Thus, the metabolic cooperation and competition among the different TME components besides the inherent alterations in the tumor cells arising out of genetic as well as epigenetic changes supports growth, metastasis, and therapeutic resistance. This review focuses on the metabolic remodeling achieved through an active cooperation and competition among the three principal components of the TME—the tumor cells, the T cells, and the cancer-associated fibroblasts while discussing about the current strategies that target metabolism of TME components. Further, we will also consider the probable therapeutic opportunities targeting the various metabolic pathways as well as the signaling molecules/transcription factors regulating them for the development of novel treatment strategies for cancer.

## Introduction

One of the important hallmarks of tumor cells is the metabolic reprogramming, where the tumor cells metabolize glucose even in the presence of abundant oxygen (aerobic glycolysis), widely referred to as the Warburg effect (1). This reprogramming is purported to facilitate the survival and growth of transformed cells by enhancing macromolecular synthesis and antioxidant defense, besides the energy production.

Tumor cells in a solid tumor coexist with different types of host cells like the fibroblasts, cells of the immune system like lymphocytes and macrophages, and the endothelial cells constituting the blood vessels besides a host of secreted factors generated by the tumor as well as non-tumor cells. Through the paracrine signaling, tumor cells constantly modify the environment that facilitates the survival and growth of the tumor, as well as provides escape from immune surveillance (2, 3). The metabolic pattern in a cell is not merely governed by the availability of substrates but is also influenced by the signaling pathways stimulated by the metabolites and the environmental factors (4). The metabolic phenotype of fibroblasts and subsets of lymphocytes within the tumor microenvironment (TME) show a considerable degree of heterogeneity (5), while their stimulation leading to proliferation and functional maturity is invariably preceded by the reprogramming of the metabolism (6, 7). It is increasingly becoming clear that the TME consisting of extracellular matrix (ECM), abnormal stroma, and altered vasculature has a strong role in shaping the metabolic phenotype of tumor cells, besides the genetic and epigenetic changes that results in the reprogramming of the cancer cell metabolism (8, 9).

Accumulating evidences strongly support the notion that a metabolic dependence exists between the tumor cells and the cells in the stroma, which show temporal and context-dependent variations that provide support to the tumor cells through the shuttling of metabolic intermediates and oxidative stress components leading to signaling changes in the tumor as well as cells in the microenvironment including stromal cells and cells of the immune network (10, 11). Current understanding of the metabolic reprogramming in tumors, including the interplay with oncogenic processes and their implications for diagnosis and developing therapeutics has been extensively reviewed and so is the diversity of the metabolic pattern in immune network and their reprogramming following stimulation (12–20). This review focuses on the metabolic reprogramming in the tumor milieu consisting of the tumor cells and cells in the microenvironment for identifying suitable targets for developing newer therapeutic approaches.

## Components of Tumor Microenvironment

In the last two decades with the emerging knowledge on TME, the understanding about the host–tumor interactions within the TME has attained new dimensions. The cellular milieu within a solid tumor consists of a myriad combination of cells, signaling molecules, and ECMs. All these form a heterogeneous medium around the tumor cells known as the tumor stroma or the TME (21, 22). The diverse array of cells within the TME originates from the surrounding host tissues and could be either hematopoietic or mesenchymal in origin. The hematopoietic cells in TME are the B cells, T cells, neutrophils, natural killer (NK) cells, and macrophages while the fibroblasts, adipocytes, endothelial cells, and pericytes are the mesenchymal component of TME. Collectively, these cells comprise up to 50% of the total mass of a solid tumor (23).

The neovasculature that develops within a growing tumor mass is also an integral structural component of the TME and is essential for the development of the pro-tumorigenic atmosphere within the solid tumor. However, the tumor vasculature is larger in size compared to their normal counterparts and hence fails to penetrate deep within the tumor tissue (24, 25). Consequently, the TME becomes progressively devoid of oxygen and energy precursors from the periphery toward the core of the solid tumor. The resultant hypoxia and the nutritional stress in turn initiates a complete metabolic remodeling in the neighboring host cells that create the classical pro-tumorigenic TME including lowering of the extracellular pH (pHe) due to H^+^ and lactate generated by hypoxic cancer cells (26, 27). Hypoxia and acidosis are thus the two most important characteristics of TME. In fact, abnormally proliferating tumor cells consume increased oxygen leading to progression of hypoxia that further produces an acidic environment by upregulating glycolysis, which in turn increases proton production and results in proton efflux through several types of acid transporters causing acidosis in the TME [reviewed in Ref. (28)]. Acidosis on the other hand suppresses glycolysis and increases mitochondrial respiration in the cancer cells (28–30). This pro-tumorigenic TME fosters tumor growth and proliferation as well as promotes metastasis, augmenting invasiveness and providing protection against immune/chemotherapeutic assaults.

## Metabolism of the Components of TME

### Metabolism of the Cancer Cells

**Figure 1:**
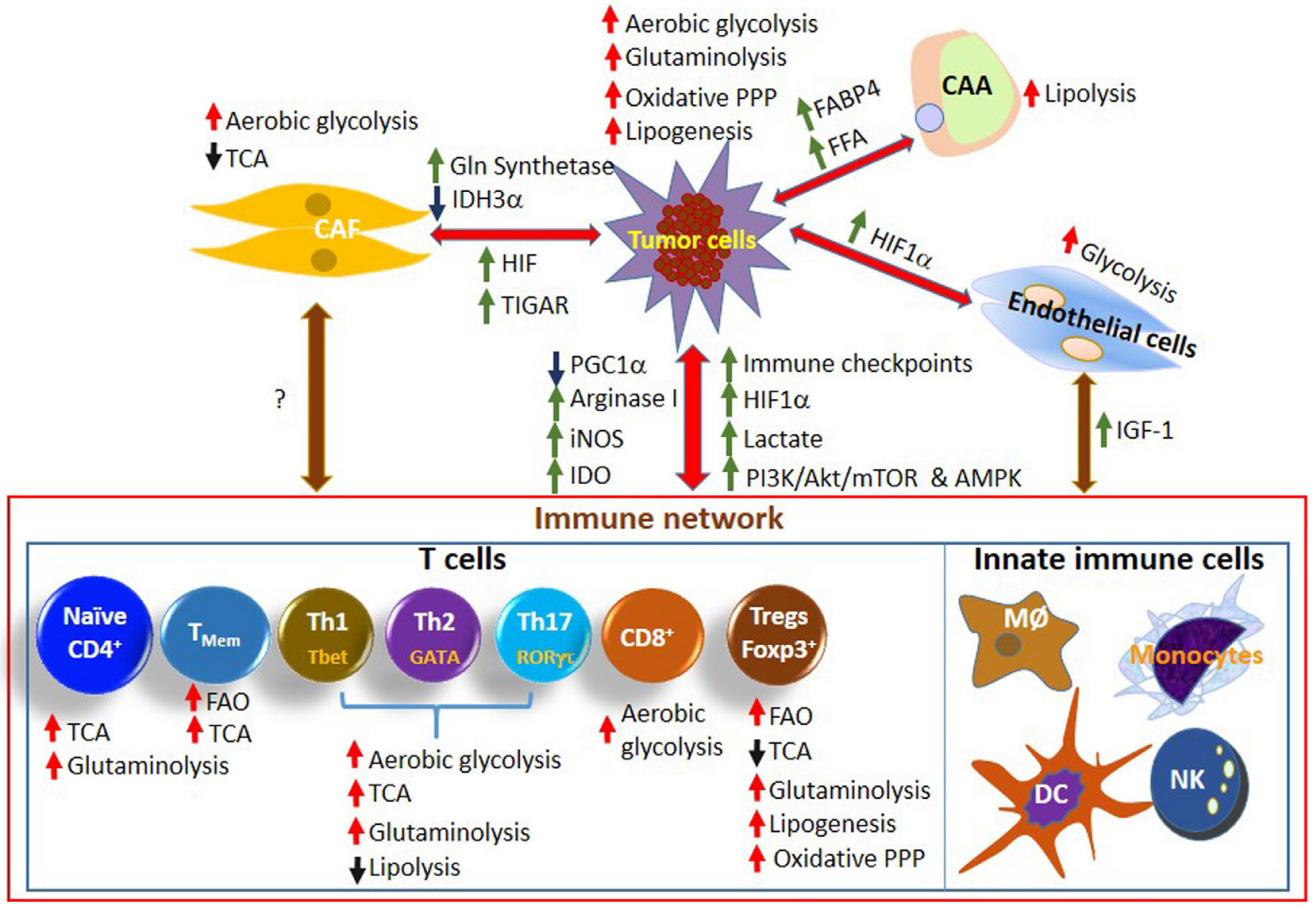
**Metabolic programing, reprograming, competition, and cooperation between cells of the TME**. The modulation of signaling pathways and metabolic enzymes as well as availability, levels, and exchange of several metabolites decide the fate of the tumor growth by affecting the functions and differentiation of various subsets of immune cells, generation of CAFs and CAAs, and proliferation of endothelial cells. FAO, fatty acid oxidation; FFA, free fatty acids; DC, dendritic cells; MØ: macrophages; TME, tumor microenvironment; CAFs, cancer-associated fibroblasts; CAAs, cancer-associated adipocytes.

The proliferation of cancer cells requires a continuous and higher rate of supply of energy as well as precursors for macromolecular synthesis. This requirement, following the malignant transformation is ensured by the reprogramming of the metabolism involving enhanced glycolysis, glutaminolysis, and *de novo* lipid biosynthesis (Figure 1) in preparation for mitosis, which also supports the maintenance of redox balance and evasion of death by apoptotic pathways (31, 32). The enhanced glycolysis, despite availability of adequate oxygen supply, metabolizing glucose to lactate was unraveled by Otto Warburg, who referred to this as “aerobic glycolysis” (1, 33) and is widely known as the “Warburg phenotype”. Metabolic reprogramming of cancer cells is a complex interplay of various signaling pathways [like phosphoinositide-3-kinase (PI3K), mammalian target of rapamycin (mTOR), Akt, PTEN, AMP-activated protein kinase (AMPK), and Notch] regulated by a plethora of transcription factors including hypoxia-inducible factor (HIF) 1α, c-Myc, and p53 (12, 34, 35). Mutation of c-Myc has also been observed in cancer cells that increases the transcriptional activities of enzymes involved in glycolysis and glutaminolysis (36, 37). Various microRNAs involved in the process of metabolic reprogramming linked to several oncogenic signaling pathways have been recently reviewed in Ref. (12).

Underlying factors that contribute to the Warburg phenotype or aerobic glycolysis include alterations in the mitochondrial functional status, upregulation of rate-limiting enzymes of glycolysis and intracellular pH regulation, loss of p53 function, and the presence of hypoxia in solid tumors (38). Hypoxia-induced HIF1 activates the transcription of several genes including the genes responsible for upregulating glycolysis such as glucose transporters (Glut), Glut-1 and 3; glycolytic enzymes, hexokinase 1/2 (HK I/II) and pyruvate kinase M2 (PKM2), and genes involved in the inhibition of oxidative phosphorylation, pyruvate dehydrogenase kinase 1 (PDK1), and lactate dehydrogenase-A (LDH-A) (39–41). High expression of HIF1α and Glut-1 are associated with poor prognosis in cancer patients (11). Furthermore, HIF1α supports energy supply to hypoxic tumor cells driving an anaerobic glycolysis by upregulating monocarboxylate transporter 4 (MCT4) that exports the lactate out of the cells (42) and influencing carbonic anhydrase IX (CAIX) to prevent the intracellular acidification (43). HIF1 also helps in reducing mitochondrial activity and reactive oxygen species (ROS) generation from oxidative phosphorylation by regulating the expression of BCL2/adenovirus E1B 19 kd-interacting protein 3 (BNIP3) and cytochrome oxidase COX-4 subunit composition (44, 45). In addition to HIF1-mediated effects, several HIF-independent pathways (such as mTOR) regulate the cancer cell metabolism (28). Under nutrient stress conditions in the TME, mTOR modulates several energy requiring processes such as mRNA translation, metabolism, and autophagy (46, 47). The upregulated glycolysis of the cancer cells and blood perfusion also influence the intracellular and pHe in the TME (48, 49). Reduced blood perfusion and preference for use of glycolysis by the cancer cells for their energy needs result in increased lactic acid production. Generation of protons during hydrolysis of ATP as well as hydration of carbon dioxide (CO_2_) by carbonic anhydrases (CA) also contributes to acidosis of the TME as both lactic acid and protons are exported out of the cancer cells over time (43, 50). Several MCTs, vacuolar type H^+^-ATPases, Na^+^/H^+^ exchangers, and other acid–base transporters are involved in the export of lactic acid and protons and their inefficient removal from the tumor interstitial space causes the acidification of the extracellular TME (28, 48). While acute acidosis decreases cancer cell proliferation and increases apoptosis (51, 52), chronic acidosis acts as a selective pressure leading to acquisition of multiple genomic mutations beneficial for cancer cell growth and adaptation (53, 54). Treatment of prostate cancer cells with acidosis is shown to reduce Akt activity (29). Therefore, reduced Akt activity may enhance the activity of Na^+^/H^+^ transporter NHE-1 causing increased proton export and cell proliferation (55, 56). Although hypoxia and acidosis in the TME are shown to induce distinct biological effects, several reports have shown both synergistic as well as antagonistic effects on tumor cell response when treated simultaneously with these stimuli [reviewed in Ref. (28)]. In cases of oral squamous cell carcinoma, proteins associated with glucose and lactate metabolism are often found to be co-localized in the hypoxic areas (57, 58) and therefore an analysis of their combined expression can be used for early diagnosis and prognosis (59).

Although regulators of various signaling pathways contributing to the Warburg phenotype would naturally be pertinent targets for designing anticancer therapeutics and adjuvant, development of effective therapies targeting this phenotype has remained a challenge till date (60). However, the enhanced glucose uptake of tumors has been widely exploited for the non-invasive detection and grading of tumors by positron emission tomography using the F-18-labeled glucose analog 2-deoxy-d-glucose (FDG) (61). It is increasingly believed that a better understanding of the mechanisms underlying Warburg effects will facilitate the design of effective therapies targeting the reprogramming of metabolism (14). Renewed interest in unraveling the mechanisms underlying the development of Warburg phenotype and its relationship with therapeutic resistance of tumors (12, 62–65) holds great promise in the future for developing novel therapeutic strategies targeting metabolic reprogramming of tumors (60).

In a rapidly proliferating tumor cell, alternative pathways of glucose metabolism, like the pentose phosphate pathway (PPP), are essential for generating important biomolecules like NADPH and ribose sugars (Figure 1). For the tumor cells, the NADPH is essential to fulfill various metabolic requirements like ATP production, lipogenesis as well as for eliminating the oxidative stress. Similarly, the ribose sugar as an integral part of the nucleotides is essential for rapidly dividing cells. In fact, a high ratio between the oxidative and non-oxidative branches of PPP is known to promote the proliferation of several types of cancer cells (66, 67). In HCT116 colon adenocarcinoma cells, regulators of cell cycle progression like CDK4 and 6 have also been found to be involved in maintaining the crucial balance between the two branches of PPP (68).

**Figure 2:**
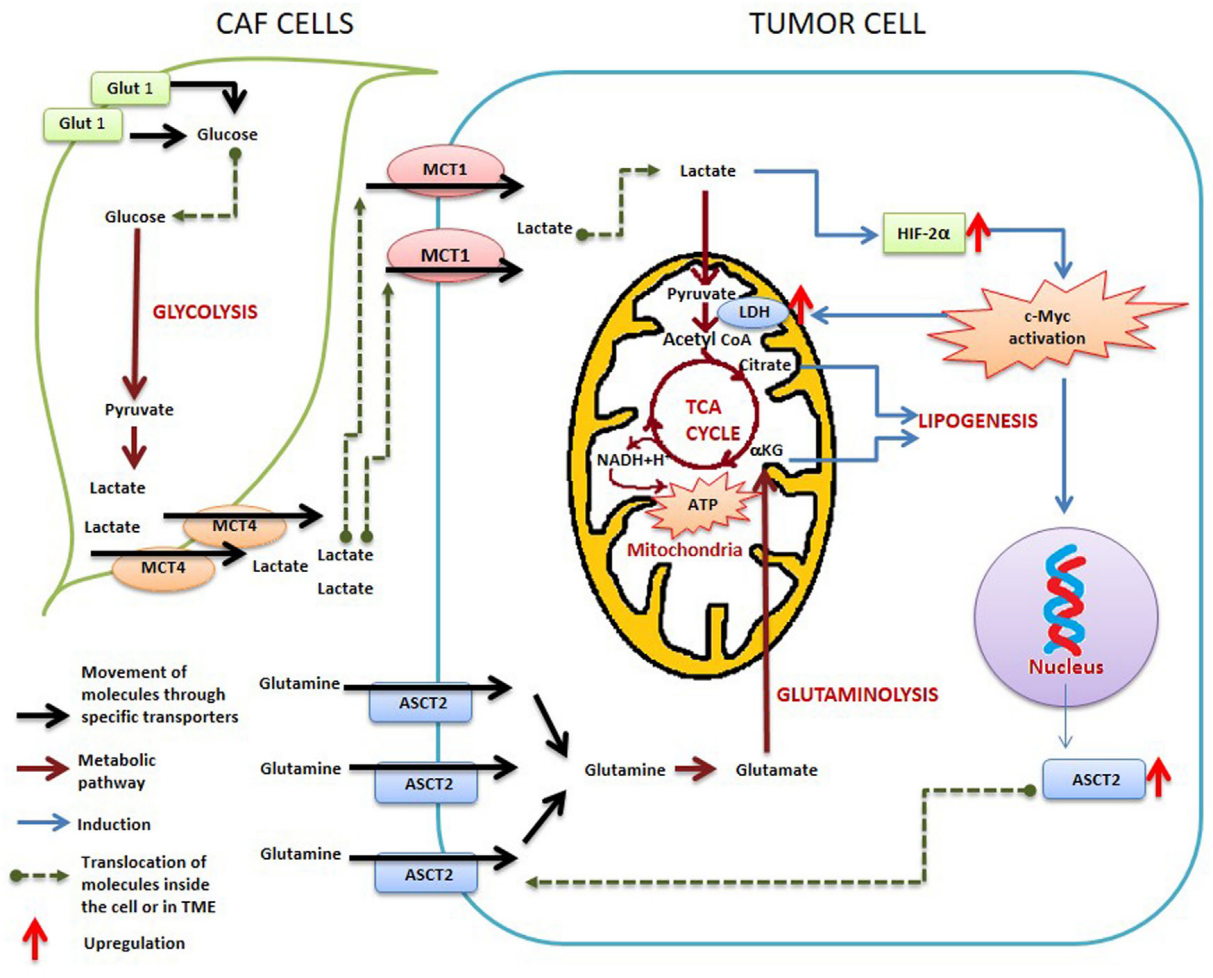
**Metabolic reprogramming between cancer-associated fibroblasts (CAFs) and tumor cells within tumor microenvironment**.

To support the overall growth, cancer cells need adequate amount of macromolecules like nucleic acids, lipids, and proteins. Highly proliferative cancer cells are associated with a strong dependency on lipid and cholesterol, which are satisfied by either enhanced uptake of exogenous (or dietary) lipids and lipoproteins or by increasing the activation of endogenous synthesis (69). Indeed, the lipid droplets consisting of cholesterol and other lipids found in some of the tumor cells are now considered as hallmarks of the degree of aggressiveness of the cancer (69). Specific lipids are now known to mediate intracellular oncogenic signaling, defense against endoplasmic reticulum stress, and interactions with cells of the TME (69). Since HIF1 inhibits mitochondrial oxidative phosphorylation, it also inhibits the fatty acid synthesis from glucose-sourced carbon as pyruvate is not utilized in the mitochondria (28, 70). Therefore, to meet the increasing demands of ATP and the lipids, growing tumor cells increase the uptake and synthesis of glutamate as an alternative carbon source. Tumor cells utilize glutamine as a nitrogen donor for essential amino acid and nucleotide biosynthesis as well as to generate α-ketoglutarate which can be channelized toward tricarboxylic acid (TCA) cycle for energy production (71, 72). Glutamine can enter the cell through glutamine transporters like SLC1A5 (ASCT2) and SLC38A5. The levels of these receptors especially that of SLC1A5 are found to be overexpressed in breast and prostate cancer cell lines and pharmacological inhibitors such as benzylserine (BenSer) and l-γ-glutamyl-*p-*nitroanilide (GPNA) or shRNA-mediated inactivation/suppression of the glutamine transporter has been found to stall the proliferation of tumor cells (73, 74) (Table 1). The uptake of glutamine in tumor cells is in turn governed by its lactate uptake as acidic TME supports activation of p53 and increases glucose 6-phosphate dehydrogenase (G6PD) and glutaminase 2 (GLS2) (75). Within the tumor cells, lactate obtained from the neighboring tumor stroma stabilizes the HIF2α which in turn activates the oncogene c-Myc and upregulates the expression of both glutamine transporter ASCT2 and glutaminase 1 (GLS1)—thus ensuring a steady flux of glutamine in the cells (76) (Figure 2). Further, in addition to the glutamine, metabolism of other amino acids such as arginine, tryptophan, glycine, serine, and branched chain amino acids (BCAAs, leucine, isoleucine, and valine) play an important role in tumorigenesis and TME (77).

**Table 1:** **Therapeutic agents (small molecules) targeting different cells of the TME and their associated metabolism**.

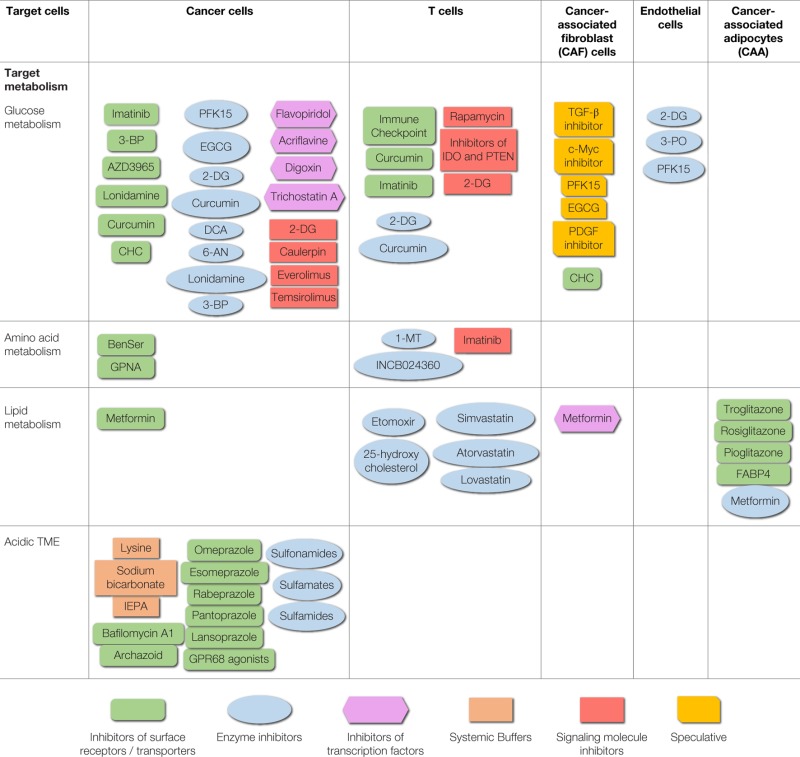

### Metabolism of the Immune Cells

#### Cells of the Immune Network

Solid TME is infiltrated by various heterogeneous immune cell types that work in a coordinated fashion against the tumor antigens (Figure 1). Their proliferation, effector function, and differentiation are regulated by several signals that are influenced by the metabolic activity. Although several types of innate immune cells such as NK cells, macrophages, and dendritic cells (DCs) play an important role in mediating the antitumor effects (Figure 1), here we are focusing more on immune functions mediated by T cells.

Transition of T cells from naïve to effector and to memory phenotype requires specific metabolic programing and reprograming to match their proliferation status and function (78). The naïve and memory T cells utilize oxidative phosphorylation to derive ATP for their needs. However, proliferating lymphocytes reprogram their metabolism and switch to glycolysis for fulfilling the energetically demanding processes of cell division and effector functions. Presence of glucose and amino acids such as glutamine is essential to support the changing demands of proliferation and biosynthesis utilizing distinct metabolic pathways (79, 80). Further, different T cell subtypes depend on different metabolic pathways for their energy needs and thus metabolism plays a key role in determining the T cell fate, differentiation, and function (Figure 1). In addition to the metabolic cooperation between different cell types, other factors such as oxygen pressure and presence/availability/levels of different metabolites affect the proper functioning of immune cells. Therefore, TME plays an important role in determining the T cell-mediated immune response as activated T cells go from an oxygen and nutrient-rich environment in the periphery to the hypoxic and nutrient-poor environment of solid tumors (13).

#### T Cell Metabolism

There are several reasons that lead to progression of cancers. Cancers that have weakly immunogenic antigens can evade killing (81). Cancers can also evade killing due to T cell dysfunction, anergy, exhaustion, senescence, or hypo-responsiveness (82, 83). Although several factors may affect the function of T cells, metabolic competition between tumor cells and T cells is now emerging as one of the major contributors for tumor escape. Like the other normal cells in the body, T cells have specific energy requirements according to their function and activation status (Figure 1). Both CD4^+^ and CD8^+^ T cells in resting state generate most of their energy using TCA cycle as they have low metabolic requirements (84). They need limited biosynthesis and oxidize pyruvate and lipids as well as amino acids for energy production. However, when the T cells are activated, they shift to glycolysis and other anabolic pathways and use the metabolic intermediates of TCA cycle to synthesize proteins, lipids, and nucleic acids (13, 85). This switching provides several advantages as it leads to rapid turnover of ATP (although aerobic glycolysis is less efficient as number of ATP molecules generated is much less than oxidative phosphorylation); decreased production of ROS; generation of metabolic intermediates needed for growth and proliferation; and accommodation of T cell survival in hypoxic environment generally present in the solid tumors [reviewed in Ref. (20)]. CD4^+^ T cells show enhancement in both glycolysis and oxidative phosphorylation upon activation, while CD8^+^ T cells may increase only glycolysis making them more sensitive to availability of glucose (5, 13). Activated T cells show increased expression of Glut-1 on their surfaces for facilitating enhanced uptake of glucose (86). Extracellular signals mediated by growth factors play a significant role in increased expression and membrane localization of the transporters. The expression of growth factors and their receptors change with the activation status of the T cells (87). For example, IL7 receptor expression increases in naïve cells, decreases in activated cells with increased dependence on IL2 and then again increases during differentiation of T cells to memory T cells (88). The change in the levels of the growth factors is reflected in change in the cellular metabolism and their withdrawal results in removal of nutrient transporters from the cell surface and decreased glycolysis among other metabolic changes (89, 90). Increase in glycolysis is generally also accompanied with increase in glutamine oxidation and decrease in lipid oxidation (13, 80) (Figure 1). Glutamine metabolism may also regulate the balance of effector and regulatory T cells (Tregs). Loss of the neutral amino acid transporter protein, ASCT2 in T cells resulted in impaired generation and function of Th1 and Th17 cells without altering Tregs generation (91). Similarly, arginine regulates the expression of components of T cell receptor (TCR) (92) and cell cycle progression in T cells (93).

The induction of aerobic glycolysis during T cell activation is dependent on the PI3K pathway (94). Downstream of PI3K pathway, Akt has been shown to affect the expression of Glut-1 and its translocation to the cell membrane (86, 95). Akt is known to control the activation status of mTOR that controls protein synthesis, mitochondrial activity, and proliferation (96, 97). Therefore, in addition to the extracellular signals mediated by several growth factors, PI3K/Akt/mTOR signaling triggered by TCR and co-stimulatory signal through CD28 play major roles in metabolic reprograming of T cells during their activation (94). mTOR upregulates c-Myc and HIF1α although only c-Myc is required for the glycolytic switch as its early upregulation is crucial in the activation process of T cells (98).

Following activation and division, T cells differentiate into different subsets that switch on distinct metabolic pathways appropriate for their function. mTOR and other signaling pathways such as Myc and HIF1α play significant roles in determining these phenotypes in effector T cells (99, 100). T helper (Th) cells; Th1, Th2, and Th17 rely more on aerobic glycolysis where mTORC1 and 2 help in deciding the metabolic phenotype while Tregs and memory T cells achieve their metabolic needs principally through fatty acid oxidation (FAO) that is controlled by AMPK (101). The decrease in dependence on glycolysis and utilization of lipid metabolism may play a role in survival advantage of Tregs and memory T cells (102, 103) (Figure 1).

### Metabolism of the Mesenchymal Cells

#### CAF Metabolism

Cancer-associated fibroblast or CAF are a group of specialized fibroblasts that is considered to be the principal non-cancerous cell type within the TME. In normal tissues, the fibroblasts remain embedded in a comparatively dormant state in the ECM. They synthesize and secrete collagen, fibrous proteins like reticulin and elastin, proteoglycans, glycoproteins, and various other components of the ECM that act as a cementing material among the cells and helps in maintaining a cohesive organ structure (104).

Within the TME, the normal fibroblasts transform into a highly synthetic, metabolically active, contractile form that resembles the “activated myofibroblasts” (105) observed in the wound site during tissue damage and repairing process. The tumor cells require the presence of such activated fibroblast or CAF in their vicinity to generate a favorable atmosphere for them. The CAFs are known to actively promote proliferation and differentiation of tumor cells as well as support angiogenesis and metastasis by promoting matrix remodeling and epithelial to mesenchymal transition (EMT) (106–108). Even the enrichment of stroma/CAFs within the tumor tissue has a direct correlation with the tumor size and a negative impact on the clinical prognosis (109)—as observed in cases of gastric signet ring cell carcinoma—indicating the profound impact of CAFs on overall tumor biology.

In recent years, growing knowledge about TME and the metabolic crosstalk between the cancer cells and the associated CAF cells have generated tremendous interest regarding the bioenergetics of the various cellular compartments of the TME. The metabolic hallmark of the CAF is their high glycolysis (Figures 1 and 2). Several studies have indicated the presence of an increased expression of MCT4 in CAF-mediating lactate efflux from them (110, 111) (Figure 2). On the other hand, in the osteosarcoma cells, an increased expression of MCT1 mediating lactate influx has been observed (112). Similarly, increased production of lactate associated with upregulation of MCT1 and 4 has been observed in CAFs associated with breast (113) and bladder (114) cancer cells. Such observations clearly indicate the dependence of the cancer cells on metabolites provided by the CAF cells.

The lactic acid present in the TME along with the hypoxic environment is also known to mediate the transformation of the macrophages from M1 to a pro-tumorigenic M2 phenotype through a HIF1α-mediated pathway (115, 116) by directly inducing M2-like gene expression (augmented expression of VEGF, Arg1, PKM2, etc.) in tumor-associated macrophages (TAMs) (116, 117). Recent studies have also suggested that the excess lactic acid produced by the heightened glycolysis observed in CAF—is one of the chief regulators that orchestrates the metabolic transformation of the different cells that reside within the TME (118) (Figure 2).

#### Endothelial Cell Metabolism

Like the cancer cells and CAFs, endothelial cells also rely on glycolysis to sustain themselves in the hypoxic TME (Figure 1). To support the cancer cells, endothelial cells also need to maintain a high degree of proliferation. The hypoxic environment of TME along with pro-angiogenic signals such as VEGF lead to the upregulation of glycolytic enzymes like glyceraldehydes-3-phosphate dehydrogenase (GAPDH) and glycolytic regulators like phosphofructokinase (PFK)-2/fructose-2,6-bisphosphatase 3 (PFKFB3) and Glut-1 thereby promoting glycolytic mode of metabolism [reviewed in Ref. (119)].

#### Metabolism of Adipocytes

Adipocytes are one of the important components of TME (120). In the normal tissue, adipocytes uptake the fatty acids, activate them, and transfer the resulting CoA derivatives to glycerol forming triacylglycerols (121). However, adipose cells need glucose for the synthesis of triacylglycerol. Most of the fatty acids formed on hydrolysis are reesterified if glycerol 3-phosphate is abundant, while they are released into the plasma if glycerol 3-phosphate is scarce because of a paucity of glucose. Thus, the glucose level inside adipose cells is a major factor in determining whether fatty acids are released into the blood (121).

## Metabolic Cooperation and Competition in the TME

Tumor microenvironment is very complex and heterogeneous where various types of cells including cancer, immune, endothelial, fibroblasts, etc. reside and interact with each other in a unique environment (122). Tumor cells are highly metabolic and other cells surrounding the tumor either compete with the cancer cells causing metabolic antagonism or support them by forming a metabolic symbiosis (15). A competition between cells of the TME occurs as demands for resources in the microenvironment are high. Tumors reprogram their metabolism in such a way that either directly supports tumor proliferation or shapes the microenvironment favoring tumor cell survival (15). For example, tumors cells are known to express and release several cytokines, lactate, and indoleamine 2,3-dioxygenase (IDO) that help in inhibiting the proliferation and function of T cells. Further, increase in HIF signaling and activation of oncogenes in the cancer cells improve their metabolic fitness resulting in deprivation of vital metabolites such as glucose and glutamine for stromal cells (15). This competition between the different cells in the TME promotes immune suppression due to the exhaustion of immune cells (82, 123). In turn, antitumor immune cells such as effector T cells and cytotoxic T lymphocytes (CTLs) reprogram their metabolism to robust aerobic glycolysis and glutaminolysis leading to metabolic antagonism with the tumor cells while the pro-tumoral immune suppressive cells such as Tregs, myeloid-derived suppressor cells (MDSCs), and M2 TAMs utilize the products generated from tumor metabolism forming a metabolic cooperation within the TME (15).

Studies in the last two decades have established CAF as one of the dominant factors that govern the proliferation of tumor cells and progression of tumor growth. CAFs appear to exert an influence on proliferation through paracrine signaling. The conditioned media from the cultures of CAFs of oral carcinoma has been found to augment the proliferation of tongue cancer cells suggesting the presence of a paracrine machinery involved in the process (124). Further, CAFs isolated from prostate carcinoma has been shown to augment the rate of proliferation of even normal prostate epithelia cells (125, 126) thus emphasizing the growth promoting influence of the CAFs. Similarly, in the TME, adipocytes present in the vicinity of the tumor undergo several functional changes to become cancer-associated adipocytes (CAA) and support growth of the tumor (120).

### Metabolic Reprograming of T Cells in the TME

Several studies have suggested that T cells become anergic or exhausted in the established tumors leading to their dysfunction and immune escape of tumors. Hypoxia and availability of various metabolites and nutrients are the two most important properties of the TME driving the metabolic reprograming in these cells.

#### Effect of Hypoxia on T Cell Metabolism

As the metabolic pattern and functionality of the immune cells are dependent on the cytosomatic cues and the partial oxygen tension of the surrounding medium, the immune cells suffer a vast transformation as they travel deeper into the hypoxic interior of the solid tumor. Hypoxia is one of the most important cues in the TME that modulates the metabolism of cancer as well as all cell types of innate and adaptive immune system thereby potentiating tumor progression. Presence of hypoxic regions in solid tumors enhances the pro-tumorigenic immune suppressive environment. HIF family of transcription factors plays a central role in the cellular responses of both tumor and stromal cells. Both oxygen-dependent and oxygen-independent regulation of HIF1α has been reported in these cells (127).

Under hypoxic conditions, HIF1α gets activated and regulates the expression of several enzymes involved in glycolysis such as LDH and PDK1 (128) and glycolysis-related genes, GLUT-1 and PFKFB3 (129). This results in increased glycolysis and decreased oxidative phosphorylation and oxygen consumption (130). Increased lactic acid production by tumor cells under hypoxic conditions inhibits the proliferation and functions of T cells of the adaptive immune system (Figure 1). Controversial role of HIF signaling has been reported in determining the differentiation of CD4^+^ naïve T cells into either Th17 or Treg cells with some reports suggesting induction and others inhibition of these cell types (131–133). HIF1α has been reported to target Foxp3 for proteasomal degradation and therefore inhibits Treg differentiation and shifts the balance toward Th17 (131). Dang et al. also showed that HIF1α shifts Th2 to Th17 differentiation by direct upregulation of IL17 gene and increased transcription of RAR-related orpha receptor γ (RORγt) (132). However, Th17 induction is shown to be accompanied with enhanced glycolysis mediated by mTOR/HIF1α signaling as upregulation of HIF1α results in increased expression of Glut-1 and therefore glycolysis in Th17 cells (134) unlike Tregs that depend on FAO for their metabolic needs. Therefore, more studies are needed to understand the role of hypoxia/HIF-mediated signaling/glycolysis in different subsets of T cell metabolism, differentiation, and function.

In addition to T cells, hypoxia has also been shown to either subvert the antitumorigenic functions toward pro-tumorigenic functions or enhance the immune suppressive functions of the cells of the innate immune system; TAMs, and tumor-associated neutrophils (TANs) (127). Hypoxic environment in the tumors promotes the polarization of TAMs toward pro-tumorigenic M2 phenotype either directly by inducing M2-like gene expression (augmented expression of VEGF, Arg1, PKM2, etc.) in TAMs (135) or due to hypoxic metabolism by tumor cells (elevated lactate levels) in HIF1α-dependent manner (116). In addition to this metabolic symbiosis between tumor cells and macrophages affecting the immune response, tumor hypoxia-mediated recruitment of endothelial cells results in interaction of these cells with M2 macrophages as they also play significant role in angiogenesis (127, 136) (Figure 1). More recently, hypoxic TAMs have been shown to upregulate the expression of REDD1, a negative regulator of mTOR hindering glycolysis and angiogenic response revealing a functional link between TAM metabolism and tumor angiogenesis (137). Cross-talk between these cells thus influences the availability of oxygen, cellular metabolism, as well as the antitumor immune response (127).

The transformation of the macrophages from M1 to M2 phenotype can be considered as the cornerstone of the metabolic immune-compromised milieu of the TME. The M1 and M2 macrophages not only differ in their immunological functions but vary greatly in their metabolic dependence as well. The M1 macrophages, providing protection against bacterial infection, depends principally on glycolysis for ATP generation but the M2 macrophages, populating the sites of healing wounds, utilize the FAO and oxidative phosphorylation for their sustenance (138) and does not compete with the tumor cells for resources in a nutritionally challenged TME. It is tempting to speculate that this scarcity of resources within the TME could also act as competitive inhibitor that quickly eliminates the glycolysis dependent, antitumorigenic M1 macrophages from the TME. The M2 type macrophages, but not the M1 type, also secrete insulin-like growth factor-1 (IGF-1), which promotes tissue regeneration (139) and angiogenesis (139, 140) hence might be involved in replenishing the TME (Figure 1).

The different types of T cells that are known to infiltrate the TME include the memory T cells, Th1, Th2, and the Th17 cells. The memory T cells are cytotoxic and are supported by the Th1 cells and their abundance is related with positive clinical outcome whereas the higher titer of Th2 and Th17 leads to poor clinical prognosis (141). Within the solid tumor, the M2 macrophages create a pro-tumorigenic atmosphere by strongly promoting the generation of the Th2 cells while actively suppressing the proliferation of antitumorigenic T cells. In fact the M2 bias along with ligands like galectin-9—secreted by the tumor cells (142)—is known to stem the proliferation of peripheral monocytes as well as induce Th1 cell apoptosis (143). Th2 along with HIF1α is also known to promote the differentiation of the Th17 subset of T cells. Th17 and its associated interleukins like IL17, IL23, IL25, etc. are reportedly involved in carcinogenesis [induce colon tumorigenesis through a STAT3-mediated pathway (144)], tumor progression (145), and subsequent negative clinical outcome (146). Similarly, it has been demonstrated that HIF1α is essential for regulation of metabolic activity in neutrophils and the absence of HIF1α resulted in drastic reduction in ATP and the killing function of neutrophils (147). Hypoxia also enhances the suppressive function of MDSCs thereby suppressing antitumor immunity. Furthermore, hypoxia is known to increase HIF signaling and upregulate HIF targets and increase the expression of arginase I causing increase in MDSC suppressor function (148).

Thus, through ECM remodeling, growth factor signaling, and evasion of immune response recruited stromal cells enhance tumorigenesis. Further, hypoxic TME results in metabolic symbiosis between hypoxic and normoxic compartments of the tumor. The products of highly glycolytic hypoxic cells such as lactate are used by normoxic cells to produce ATP through oxidative phosphorylation leading to sustained metabolic fitness of the tumor (18).

#### Metabolites and Nutrients Availability

Both cancer and activated immune cells depend on aerobic glycolysis for their energy needs as both are highly proliferating. This results in a competition for available nutrients to meet their energy and biosynthetic requirements influencing the T cell metabolism affecting their function, proliferation, as well as differentiation. Tumor cells by utilizing more glucose and glutamine create a state of nutrient deprivation for the T cells (16). This nutrient deprivation may result in T cells anergy, exhaustion, and death thereby compromising their effector functions (16). A decrease in the glucose concentration due to increased consumption by tumor cells has been shown to metabolically restrict T cells (16, 149). This leads to decreased mTOR activity, glycolytic capacity, interferon-γ (IFN-γ) production, and cytolytic activity *via* production of granzyme and perforin in T cells resulting in tumor progression (150–152). Similarly, depletion of glutamine, which is required for replacing the metabolites removed from TCA cycle for biosynthesis, has been shown to impair the function of T cells (153). Tumor cells also change themselves, for example, by oncogenic mutations resulting in continuous activation of growth and division (154). Furthermore, there may be an increase in the immunosuppressive factors produced either by cancer or other cells in the TME. Growth factor withdrawal also affects the general metabolism (87) because it results in removal of nutrient transporters from the cell surface and decreased glycolysis (89, 90, 155). Further, deprivation of growth factors leads to a decrease in availability of mitochondrial substrates for oxidative phosphorylation, changes in the mitochondrial morphology, and depolarization of the mitochondrial membrane (89, 90, 155). These metabolic changes are followed by release of pro-apoptotic factors and commitment to cell death by apoptosis (156). Recently, it has been demonstrated that tumor-infiltrating T cells have persistent loss of mitochondrial function and mass in a TME-specific effect as signals in TME can repress T cell oxidative metabolism resulting in effector T cells with modified metabolic needs that cannot be met (157). For example, tumor-infiltrating T cells showed a loss of peroxisome proliferation-activated receptor (PPAR)-gamma coactivator 1α (PGC1α) that programs mitochondrial biogenesis (157) (Figure 1). Reprogramming of the metabolism through enforced expression of PGC1α reinvigorated the function of tumor-specific effector T cells resulting in improved intra-tumoral metabolic and effector functions (157).

More recently, it has been recognized that in addition to T cell exhaustion, availability of certain metabolites such as lactate, tryptophan and arginine-related metabolites, and phosphoenolpyruvate (PEP) can modulate the activity of tumor-infiltrating lymphocytes (TILs) (158). Ho et al. discovered a new role for the glycolytic intermediate PEP in controlling the activity of effector T cells (123). They found that PEP regulates the amplitude of TCR-mediated Ca^2+^ flux and nuclear factor of activated T cells (NFAT) activation by repressing activity of sarco/ER Ca^2+^-ATPase (SERCA) in intra-tumoral CD4^+^ T cells. By overexpressing PEP carboxykinase 1 (PCK1) in T cells that leads to increased production of PEP, stronger antitumor responses were observed. Similarly, a secondary role has been discovered for glycolytic enzyme, GAPDH in regulating the effector functions of T cells (153). GAPDH inhibits IFN-γ mRNA translation when glycolytic rates are low (153). Further, lactic acid production and consequent acidification in the TME are shown to inhibit proliferation and cytokine production in CTLs (159, 160). Buffering of lactic acid *in vitro* (159, 161) or *in vivo* using proton pump inhibitor, Esomeprazole (161) resulted in complete reversal of suppressive effects of lactic acid in CTLs. By suppressing PI3K/Akt/mTOR pathway, lactate can also inhibit glycolysis (29). Lactate-mediated acidification and low pH in the TME can regulate macrophage polarization and induce arginase I leading to arginine depletion and inhibition of T cell proliferation and activation (116, 162) (Figure 1). Since Tregs prefer oxidative metabolism, it is anticipated that excess lactate can be utilized by Tregs preferentially compared to effector T cells (16). Increased lactic acid also inhibits monocyte-derived DC differentiation and activation (163) although it does not affect Tregs (101). Acidosis in the TME is also shown to stimulate activity of neutrophils (164) while repressing the functions of NK cells (165, 166). Succinate and succinate receptor, G protein-coupled receptor 91 (GPR91) have been shown to sense immunological danger (167, 168) inducing inflammation, which may be of consequence as succinate levels may drop due to decreased flux through the TCA cycle in the mitochondria.

In addition to glycolysis, amino acid metabolism particularly l-arginine and tryptophan catabolism is also dysregulated in cancers (71, 169). Activity of two important enzymes in arginine metabolism, induced nitric oxide synthase (iNOS) and arginase (ARG), is upregulated in several cancers (170, 171) (Figure 1). These enzymes create toxic reactive nitrogen species (RNS) such as peroxynitrite that is shown to induce apoptosis in lymphocytes and negatively affect T cell-mediated immunity in the tumors (172–174). Increased RNS can modulate tyrosine phosphorylation of several proteins leading to downregulation of membrane receptors such as CD4, CD8, and chemokine receptors in T cells (175). Further, enhanced l-arginine metabolism could also be responsible for anergic state of lymphocytes in the TME as addition of inhibitors of ARG and iNOS results in activation of CTLs (176). Altered l-arginine metabolism in the tumor could also lead to local arginine deficiency affecting protein synthesis in T cells (177, 178) and therefore impairing the cytokine production and effector function (179). Many tumors are known to lack an enzyme argininosuccinate synthetase 1 and therefore depend on exogenous arginine for growth (180). Tumor-associated myeloid cells (TAMCs) such as MDSCs, macrophages, monocytes, and neutrophils provide arginine to the tumor cells (181). Further, MDSCs in the TME express high levels of arginase-1 and lower arginine levels lead to inhibition of antigen-specific T cell responses due to TCR expression inhibition (178). MDSCs also sequester cysteine resulting in amino acid deprivation and inhibition of T cell activation (182).

Similar to l-arginine, local depletion of tryptophan results in T cell apoptosis and anergy (183). Increased IDO enzyme activity in the tumor cells results in accumulation of kynurenine and its derivatives and tryptophan depletion that inhibit proliferation and activation of immune cells (184) and is associated with extensive disease and immune suppression (183, 185–187). IDO enzymes are intracellular and are not secreted; however, the metabolic effects of these enzymes are not restricted to the expressing cells (183). The neighboring cells present in the TME respond to the depleted levels of tryptophan and also the secreted kynurenine thereby efficiently inhibiting the proliferation and activation of the cells (183, 184). IDO expression is also upregulated when cytotoxic T-lymphocyte antigen-4 (CTLA-4) expressed on Tregs binds to CD80 and CD86 on DCs inducing tumor antigen tolerance (188). With respect to the amino acid metabolism, a competition between tumor and immune cells also exist for serine and glycine utilization to synthesize building materials for cell growth and proliferation (189). Recently, it is also suggested that cancer and T cells may share similar requirements for BCAA catabolism that regulates the mTOR signaling (77).

To generate an intracellular source of nutrients under nutrient-limiting conditions in the TME, induction of autophagy has been observed (190). Furthermore, phosphorylation and activation of a protein kinase unc-51 like kinase 1/2 (Ulk1/2) by AMPK is shown to connect energy sensing with autophagy (191). If the metabolic stress is extensive then it may lead to T cells apoptosis (192).

In contrast to the activated effector T cells, nutrient-restrictive TME does not affect the immunosuppressive functions of Tregs (19), since Tregs preferentially utilize lipid beta-oxidation and have high levels of activated AMPK (101, 193) (Figure 1). Indeed, activation of AMPK signaling by treatment with metformin resulted in reduced T effector cells and increased Tregs (101, 194). Further, the metabolic products of tumor cells such as lactate and kynurenine are utilized for Treg differentiation (195). Furthermore in the TME, TGF-β (196) and chemokines such as CCL22 (197) are present abundantly that help in the differentiation and recruitment of Tregs. Indeed, the increased presence of Tregs in the solid tumors has been associated with poor prognosis (198). Recently, it has been shown that Tregs under different inflammatory conditions change their metabolic preferences leading to modulation of their proliferation and suppressive functions (199). Foxp3 decreases Glut-1 expression and glycolysis in Tregs increasing their suppressive function, while toll-like receptor (TLR)-mediated signaling enhances the expression of Glut-1 and glycolysis resulting in a decrease in their suppressive functions (199). Reduced glucose and or elevated lactate in the TME may favor the mitochondrial oxidative metabolic pathways in Tregs promoting their suppressive functions.

#### Immune Checkpoints

In addition to the availability of nutrients, the capacity of T cells to internalize and utilize these nutrients is one of the important mechanisms regulating the T cell activation (91, 200, 201). Upregulation of HIF1α, c-Myc, and PI3K/Akt/mTOR signaling following T cell activation play key roles in nutrient transport by promoting expression of glycolytic and anabolic genes including nutrient transporter, Glut-1 (91, 98, 132, 134, 201–203). Immune inhibitory checkpoint signals such as CTLA-4 and programmed death receptor 1 (PD-1) and their ligands are shown to modulate one of these signaling pathways (204) (Figure 1). By sequestration of CD28 ligands, CTLA-4 can inhibit CD28-mediated activation of Akt (86, 205) and similarly, PD-1 can reduce c-Myc expression and PI3K/Akt/mTOR signaling (206–209) resulting in reduced Glut-1 expression, glucose uptake, and aerobic glycolysis in activated T cells.

PD-1 and CTLA-4 can also promote Treg cells (210, 211) although they are Glut-1 independent as they depend more on oxidative phosphorylation (101, 134, 200). In fact, it has been observed that the tumor samples obtained from cancer patients comprise increased number of immunosuppressive Tregs and cytokines as well as increased expression of CTLA-4 and PD-1 and their ligands (212–214). However, as HIF1α is known to interact with CTLA-4 and its receptors, HIF-mediated blockade of CTLA-4 was shown to reduce the frequency of Tregs in the tumor (215). At the same time, HIF1α is associated with immune escape involving other mechanisms such as shedding of cell surface immune checkpoint regulators like MIC1 thus causing resistance of tumor cells to NK cell attack (216, 217). Since CTLA-4 and PD-1 are highly expressed on exhausted T cells and expression of their ligands on the tumor cells inhibits PI3K/Akt/mTOR signaling and the upregulation of glucose and glutamine metabolism (204), T cells may not be able to reprogram their metabolism correctly thereby severely affecting their functions (Table 1). Increased expression of PD-1 on tumor-infiltrating T cells is also associated with reduced ability to differentiate into memory T cells (218). Further, many cancers express higher levels of PD-L1 or PD-L2 and have PD-1^+^, exhausted T cells in their environment (219). Furthermore, co-localization of HIF1α and PD-L1 in tumors has been shown to be associated with worse prognosis (215, 220). A link of HIF1α with PD-L1 is demonstrated as HIF1α is shown to bind to hypoxia-response element of the PD-L1 promoter (221). Recently, an unexpected role of PD-L1 in regulating tumor cell metabolism is reported that suggests that PD-L1 can have direct effects on cancer cells (82). Since PD-L1 promotes Akt/mTOR activation and glycolysis in tumor cells, it is suggested that checkpoint blockade therapy may correct the metabolic competition-mediated nutrient availability imbalance between T cells and tumor cells through a direct effect on the tumor cells (82) (Figure 1; Table 1). Since improved clinical response and survival has been obtained with checkpoint blockade antibodies, it will be useful to explore the detailed mechanisms by which these antibodies modulate Akt/mTOR and HIF1α pathways as well as their effects on the nutrient availability and immune cell metabolism in patients.

### Metabolic Reprograming of CAFs

As the vasculature within a solid tumor is considered to be larger and “abnormal” compared to their normal counterparts (25), they are considered to be comparatively less efficient. Consequently, the supply of energy precursors like glucose and oxygen within the bowels of solid tumor becomes understandably limited and soon a nutrient-depleted/hypoxic environment is generated within the core of the solid tumor. Hence, with the increase in mass, the tumor cells become more and more metabolically dependent on surrounding fibroblast cells to provide them with high-energy metabolic intermediates essential to fuel the proliferation of the tumor cells. This requires an enormous metabolic remodeling in the CAFs in terms of glucose metabolism and they turn into the metabolic cattle of the tumor cells providing the later with energy precursors even at the cost of self-destruction through autophagy and mitophagy (222–224).

#### Reprograming of Glycolytic Pathways

The predominantly glycolytic nature of the CAFs has been well established and it is believed that the carcinoma cells “corrupt” the associated stromal fibroblasts and transformed them to the hyper-synthetic CAF (225). While proposing their “Reverse Warburg Hypothesis” in 2009, Lisanti and coworkers showed that the lysate of stromal cells from breast cancer patients with poor clinical outcome was associated with a considerable upregulation in the expression profile of glycolytic enzymes even under normoxic conditions (226) and lactate generated by glycolytic CAFs could be used by cancer cells through respiratory metabolism indicating that the high rate of glycolysis in CAF constitute the cornerstone of the metabolic rewiring occurring in CAF/TME (Figure 1). A loss of BRCA-1 and caveolien-1 was also reportedly observed with high glycolysis (227). However, the molecular association between a tumor suppressor gene and/or a membrane scaffolding protein with glycolytic pathway/regulatory enzymes still remains unclear. Similar metabolic shift toward glycolysis has been observed in CAFs isolated from several tumor types (228–230). An active lactate shuttle plying between the tumor cells and their associated CAFs have also been reported in several independent studies (231). In fact, a high expression of lactate transporters MCT4 and 1 and their associated protein like CD147 has been considered as a hallmark of hypoxia within TME (232–234) that shows significant correlation with tumor progression and negative clinical outcome (Figure 2). In addition to CAFs, acidic TME is also shown to modulate other stromal cells such as vascular endothelial cell inflammation and angiogenesis (28, 235, 236).

Recently, it has been reported that downregulation of isocitrate dehydrogenase 3α (IDH3α) in CAFs through a TGF-β or PDGF-based pathway might be the key factor that tips the balance toward glycolysis (237). It has also been suggested that downregulation of IDH3α lowers the level of α-ketoglutarate in the cell leading to low fumarate to succinate ratio. This imbalance in the relative abundance of TCA cycle metabolites leads to HIF1α stabilization and augment glycolysis (237). HIF1α-mediated high expression of MCT4 has been reported in pancreatic ductal carcinoma-associated CAFs indicating an active lactate transport within tumor stroma (234).

The identification of factor/s that alters glycolysis in tumor cells remains still elusive. It has been recently reported that the biphosphatase TP53-inducible glycolysis and apoptosis regulator (TIGAR) might hold the key for this metabolic reprogramming as overexpression of TIGAR in the breast carcinoma cells boosts the ATP production and glutamine uptake in tumor cells as well as pronounced glycolytic parameters in associated CAFs (238) (Figure 1). Overexpression of TIGAR has also been found to increase proliferation, while catalytically inactive TIGAR suppresses the tumor proliferation in carcinoma cells (238), thus reemphasizing the importance of metabolic symbiosis in tumor progression.

Activation of oncogenes and tumor suppressor genes has also been implicated in metabolic remodeling of TME. For example, within growing lymphoma cells, c-Myc was found to induce the overexpression of glycolytic enzymes like LDH-A and glucose transporters like Glut-1 and thereby maintained a glycolytic flux (239). The tumor suppressor gene p53 is known to maintain the cytochrome *c* oxidase complex through synthesis of the cytochrome *c* oxidase 2 (SCO2) protein. Hence loss of p53, as seen in majority of cancer cells, leads to a loss of functional cytochrome *c* oxidase complex/mitochondrial respiration promoting a higher rate of glycolysis in cancer cells (240). Along with SCO2, loss of p53 has also been implicated in the higher expression of TIGAR thus facilitating the metabolic symbiosis in the TME (241). Since these observations were made in homogenous cultures of tumor cells *in vitro*, it will be interesting to see if similar mechanisms are involved in bringing about the metabolic reprogramming in CAF cells present within the TME.

#### Reprograming of Glutamine-Mediated Metabolic Pathways

In addition to reprograming of glycolytic pathways, it is suggested that tumor cells might also induce glutamine addiction in the neighboring CAFs and TAMs. TAMs isolated freshly from glioblastoma exhibit a significantly high expression of glutamine synthetase—an enzyme that can convert the intracellular glutamate to glutamine which in turn could be supplied to the tumor cells to promote the latter’s proliferation (242). Glutamine deprivation has been observed to induce autophagy in tumor cells to supplement the intracellular glutamine level, while suppression of autophagy along with glutamine deprivation causes apoptotic cell death. Amelioration of these effects was observed with the addition of α-ketoglutarate (243). This clearly indicates that in the tumor cells, like glucose, glutamine basically acts as an anaplerotic energy precursor essential for running the TCA cycle (243). In line with this, CAFs isolated from primary pancreatic ductal adenocarcinoma have been recently shown to be more susceptible toward glutamine withdrawal compared to glucose deprivation (234) (Figure 2).

Taken together, these observations suggest that within the TME while glucose-6-phosphate/pyruvate/lactate generated through glycolysis and TCA cycle intermediates like fumarate, oxaloacetate, or citrate are sequestered toward generating membrane lipids, proteins, or nucleotides for the rapidly proliferating tumor cells, ATP production greatly depends on the conversion of glutamate to α-ketoglutarate that keeps the TCA cycle functional.

### Metabolic Reprograming in Cancer-Associated Adipocytes

In recent years, a characteristic pattern of lipid deposition has been unraveled in cancer cells with the help of advanced imaging technologies like Raman scattering microscopy (69). Lipid deposition has been shown to be increased in malignant and metastatic cells of breast cancer compared to their non-malignant counterpart (244). Lipids are a heterogeneous class of biomolecules which includes triglycerides, phospholipids, and cholesterols. While triglycerides are the principal storage molecule in animal body, the latter two are the integral component of the plasma membrane. Hence, it is reasonable to expect that proliferative cells like the tumor cells will have high deposition of lipid droplets. This aggressive deposition of lipids in tumor cells is achieved as a result of reprogramming of the lipid metabolism in the TME by upregulating the lipid biosynthetic machinery and/or by promoting lipolysis in adipocytes.

The pro-tumorigenic effect of the lipid molecule is evidenced by the observation that tumor cells often metastasize in the vicinity of adipocytes or in lipid rich milieu (69). In this regard, adipokines like IL8 have been reported to provide the cytochemical cue that directs the cancer cell toward a lipid rich “soil” (245). In the vicinity of tumor cells, adipocytes undergo several functional changes supporting the tumor growth and thereby transforming into CAAs (120). These changes include increased secretion of inflammatory cytokines, proteases, etc., dedifferentiation, and delipidation leading to fewer lipid droplets in the adipocytes (120). In the last few years, CAAs have emerged as one of the factors that closely promote proliferation of tumor cells, which involves various mechanisms. Soluble factors from adipocytes have also been implicated to promote breast cancer by activating Akt through phosphorylation and upregulating genes involved in cell adhesion, matrix remodeling, and angiogenesis (246). Similarly, IGF-1 released by the human adipocytes is known to promote proliferation of MCF-7 cells. The level of IGF-1 has been found to be greatly amplified in the presence of fatty acids (247) and thus could be speculated to be the link between obesity and higher cancer risk. Fatty acids provided by the adipocytes is suggested to be the energy source that fuel the metastasis of breast cancer (248) as well as induce autophagy to promote proliferation in colon cancer (249). Also the increase in the levels of fatty acid-binding proteins (FABP)—a family of protein involved in transporting free fatty acid—in several cancers like breast, prostate, ovarian, and colorectal carcinoma [reviewed in Ref. (250)] indicate the existence of an active sequestering of fatty acid occurring between the tumor cells and CAAs. An import of free fatty acids molecules to tumor cells has been reported in several types of cancers including ovarian and prostate carcinomas (245, 251). Hence the presence of CD36, an integral membrane protein associated with the import of fatty acid to the interior of the cell, has often been equated with high rate of metastasis and poor prognosis (252, 253). However, the regulation of cross-talk between the adipocytes and the cancer cells leading to the mobilization of the fatty acid has not been elucidated so far.

Lipid molecules, in addition to being a carbon sink, are also energy-rich molecules that can support the proliferation of the tumor cells in the nutrition-deprived interior of the solid tumor. CAAs thus supply energy to cancer cells through fatty acids as cancer cells induce metabolic alterations in the CAAs like increased activity of hormone-sensitive lipases that results in increased production of fatty acids from CAAs, which is then used by cancer cells (120). Indeed, certain tumors like prostate cancer have been reported to rely less on glucose metabolism (254, 255) but depend mostly on FAO for energy production (256). Simultaneously, lipid biosynthesis generates NADP^+^ which can act as an alternative acceptor for the terminal electron in electron transport chain (ETC) in the hypoxic TME (255). The NADP^+^ can also act as a substitute for NAD^+^ during glycolysis (70). Thus, lipid biogenesis not only ensures the sustenance of the ETC/ATP production but also maintains the high glycolytic flux in the TME.

## Therapeutic Targeting

One of the important considerations in therapeutic targeting of metabolism for cancer therapy is the similar requirements for anabolic metabolism by both cancer and activated T cells/stromal cells. Therefore, identification of targets, metabolites, metabolic enzymes, metabolic pathways that are differentially expressed/utilized/regulated in cancer and other stromal cells in the microenvironment is essential to avoid unintended effects on the function of stromal cells. Furthermore, this therapeutic targeting should result in increased antitumor effects of T effector cells, increased generation of memory cells, and reduced immunosuppressive functions of Tregs.

### PD-1/PD-L1/CTLA-4 Signaling

On activation, T cells reprogram their metabolism to aerobic glycolysis and glutaminolysis but PD-1 signaling suppresses Akt/mTOR pathway (204, 257) thereby impairing the metabolic reprograming and promoting the beta-oxidation of fatty acids (214). Thus, antitumor effects of anti-PD-1 therapy will also be mediated by re-engagement of aerobic glycolysis by TILs through elevated expression of Glut-1 and glycolytic proteins (Table 1). In fact, effects of anti-PD-1 therapy were abrogated in the presence of rapamycin (257). In addition to PD-1/PD-L1 signaling-mediated effects on the TILs, PD-L1 expression on the cancer cells has been shown to mediate cell-intrinsic signaling through PI3K/Akt/mTOR pathway leading to enhanced glycolysis in the cancer cells (82). Thus, metabolic reprograming both in cancer and immune cells is one of the important reasons for PD-1/PD-L1 blockade-mediated therapeutic effects (Table 1). Similar to PD-1, CTLA-4 also inhibits increased glucose metabolism following T cell activation, which is vital for naïve T cells transitioning to T effector cells (204, 205). Therefore, effects of anti-CTLA-4 antibodies in tumor therapy could also be partially mediated due to their effects on the glycolytic metabolism (Table 1). On the other hand, non-specific pharmacological/chemical inhibitors of glycolysis like 2-deoxy-d-glucose (2-DG) could be more effective as they can modify glycolysis both in cancer cells as well as the T cells, although the consequences may not be identical in both the cell populations depending on the nature of metabolic patterns in different subsets of T cells. Indeed, recent studies from our laboratory have shown that a combination of systemically administered 2-DG with focal irradiation of the grafted Ehrlich ascites tumor in mice shows selective lympho-depletion coupled with differential activation of different Th cells and polarization of macrophages to M1 phenotype that strongly correlates with the local tumor control (258, 259) (Table 1). Since both CTLA-4 and PD-1 block glycolysis, checkpoint blockade will also enhance effector T cells while potentially inhibiting Tregs as they are dependent on FAO for their metabolic needs (214). Strategies that affect the signaling mediated by other surface receptors such as P2X7 and A2AR using administration of NAD^+^ and A2AR agonists, respectively, have been shown to deplete Tregs (260, 261).

### HIF1α Signaling

Hypoxia-inducible factor 1α controls several genes involved in glucose and lactate transport and glycolysis, such as Glut-1, MCT1, and MCT4 (11, 129). In addition, HIF1α signaling also affects pH stabilization and angiogenesis thereby affecting the tumorigenesis and metastasis (11). Therefore, modifiers of lactate transport such as inhibition of MCT1 with alpha-cyano-4-hydroxycinnamate (CHC) has been shown to induce a switch from lactate-fueled respiration to glycolysis leading to retarded tumor growth by selectively killing hypoxic tumor cells (262) (Table 1). Such a strategy may also affect the polarization of TAMs (115, 116) as well as the metabolic symbiosis between CAFs and cancer cells (118) in a HIF1α-dependent manner. Further, inhibition of HIF1 transcription by flavopiridol (263), dimerization and synthesis by acriflavine and digoxin (264, 265) and induction of HIF1 degradation by trichostatin A, a histone deacetylase inhibitor (266) have been investigated as therapeutic approaches (Table 1).

Hypoxia-inducible factor 1α signaling plays a crucial role in regulating the immune response. However, both positive and negative regulatory effects of HIF1α on T effector cells have been demonstrated. Although activating HIF1α pathway in mouse melanoma cancer cells resulted in prevention of T effector cell exhaustion even in the presence of continuous antigen exposure (267), more studies are required before using HIF1α activators to enhance the T cell-mediated responses. In addition to effects of hypoxia on immune response, hypoxia also affects angiogenesis. Anti-angiogenic therapy of cancers generally by VEGF blockade results in increased hypoxia due to metabolic reprograming that leads to tumor aggressiveness and metastasis (268). It has been shown that re-expression of semphorin 3A in cancer cells improves the cancer tissue oxygenation and reduces the anti-angiogenic therapy-induced activation of HIF1α leading to enhanced therapeutic effects (269).

### PI3K/Akt/mTOR and AMPK Pathway

Increasing memory T cell prevalence has been observed with different compounds that affect PI3K/Akt/mTOR and AMPK signaling. Blocking of glycolysis by 2-DG, a hexokinase inhibitor resulted in increased AMPK activity leading to negative regulation of mTOR and Foxo1 and enhanced CD8^+^ T cell-mediated antitumor effects (270). Treatment with metformin also resulted in increased AMPK activation and memory T cell generation (271), which could be due to its effects on mTOR signaling (272) or miR33a upregulation that is responsible for downregulation of c-Myc expression (273). Rapamycin, an inhibitor of mTORC1, is shown to exert multiple effects on T cell metabolism (Table 1). It reduces glycolysis and increases lipid peroxidation through mTOR inhibition, enhances the formation of T memory cells (274), inhibits T-bet, a Th1-promoting transcription factor (275), and induces autophagy (276). However, since immunosuppressive effects of rapamycin have been reported (277), more investigations are required to determine the long-term antitumor effects of rapamycin. Since induction of Tregs has been observed in response to apoptotic tumor cells in an IDO-dependent manner, pharmacological inhibition of either IDO or PTEN resulted in loss of Foxo3A, a target of Akt as well as destabilization of Tregs causing rapid tumor regression (278) (Table 1). Further, several rapalogs such as temsirolimus and everolimus have been shown to exert anticancer effects (Table 1) although upregulation of PI3K/Akt pathway following treatment with rapalogs remains a matter of concern necessitating the deployment of combination strategies to inhibit this response (28, 279–281).

### Use of Metabolic Reprograming to Manipulate Metabolites and Metabolic Enzymes

#### Targeting Glucose Metabolism

Glycolytic metabolites like PEP act as sensors for availability of glucose in the environment and can modulate the important signaling pathways regulating the effector functions of the T cells (123). Further, glycolytic enzymes such as GAPDH also have additional role as metabolic checkpoint regulators (153). Therefore, manipulating and reprograming the metabolism in T cells by changing the levels of these metabolites and metabolic enzymes to modulate their specialized functions can be used in adoptive cell therapy (ACT) as well as an adjunct form of immunotherapy. Indeed, overexpression of either PCK1 or PGC1α in T cells has been shown to result in stronger antitumor responses emphasizing the potential of ACT where the expression of metabolic enzymes is modulated (123, 157).

Inhibition of key enzymes involved in glycolysis is one of the important strategies being considered for cancer therapy. The enzymes like hexokinase—a molecule that is involved in several pathways of carbohydrate metabolism—are emerging as one of the determinants of cancer prognosis and inhibition of hexokinase appears to be pivotal in predicting the outcome of cancer therapeutics (282, 283). 2-DG is an inhibitor of glycolysis that competitively inhibits hexokinase through product inhibition due to the accumulation of 2-DG-6-phosphate (2-DG-6-P), which is not metabolized further causing the metabolic block in the form of reduction in ATP from glycolysis and NADPH from PPP (284, 285) (Table 1). Selective sensitization of tumor cells to radiation and chemotherapeutic drugs by 2-DG arises from differential modifications of multiple damage response pathways in tumor and normal cells. This includes depletion of energy, disturbed redox balance, and altered N-linked glycosylation leading to unfolded protein responses (UPR), collectively resulting in changes in the gene expression and phosphorylation status of proteins involved in signaling, cell cycle control, DNA repair, calcium influx, and apoptosis (286). Studies with animal tumors have shown enhanced local tumor control without significant damage to the normal cells (and tissues). Phase I–III clinical trials with a combination of 2-DG and hypofractionated radiotherapy in malignant glioma patients have shown excellent tolerance with minimal toxicity and moderate survival benefits with significantly improved quality of life (287–290).

In addition to the direct effects of 2-DG on the cancer cells, systemically administered 2-DG together with focal irradiation of the tumor has been shown to activate antitumor immunity in the peripheral blood both in terms of increase in the levels of innate and adaptive cells and decrease in B cells, where a decrease in the CD4^+^ naïve T cells was paralleled by the increase in CD4^+^-activated T cells (258). This was also associated with a shift from Th2 and Th17 to Th1 in the form of cytokine and switching of antibody class, which appears to be mainly due to the selective depletion of induced T regulatory cells (CD4^+^CD25^+^FoxP3^+^ CD39^+^FR4^+^GITR^+^CD127^−^) in blood, spleen, lymph node, and the tumor (258). This appears to result in the immune activation in the periphery, secondary lymphoid organs, and massive infiltration of CD4^+^, CD8^+^, and NK cells in the tumor, which correlates well with the tumor control (258). More recent studies have shown that 2-DG in combination with tumor irradiation polarizes splenic macrophages toward M1 type *in vivo* as well as *in vitro (*RAW 264.7) that correlated well with enhanced local tumor control (259). Clearly, effects other than glycolytic inhibition like UPR response (due to altered N-linked glycosylation) and HIF1α signaling appears to be involved in the immune activation by 2-DG, which needs further studies to provide more insight (258, 259) (Table 1).

Glycolysis is associated with the activation of normal lymphocytes, i.e., the lymphocyte activation dogma (291). Interestingly, immune activation has been reported in tumor-bearing mice following systemic administration of 2-DG combined with focal irradiation of the tumor, which appears to be out of tune with the dogma, although lympho-depletion was seen 1 day after the administration (258). Interestingly, the tumor response appears to be determined by the changes in the immune status seen soon (1 day) after the treatment, suggesting that these indicators of alterations in the immune status can also serve as surrogate markers of tumor response to the combined treatment involving 2-DG.

Glycolytic inhibitors other than 2-DG have also been evaluated for their potential to influence the therapeutic response. For example, complete remission has been observed in a patient with relapsed non-Hodgkin’s lymphoma following treatment with sodium dichloroacetate (DCA) that targets PDK1 thereby reducing lactate production (292) (Table 1). Further, 6-aminonicotinamide (6-AN) has been used to inhibit the glycolytic shunt into PPP by inhibiting G6PD (293, 294) (Table 1). A higher degree of radiosensitization has been reported by a combination of 2-DG and 6-AN in both cancer cells and *in vivo* in Ehrlich ascites tumor-bearing mice (295–297). PFK—and its regulatory molecules—are also of particular interest as plausible targets for cancer therapy. PFKFB3 is known to synthesize fructose 2,6-bisphosphate (F2,6P2) which acts as an allosteric activator for PFK-1. Hence, small molecule inhibitors of PFKFB3, like PFK15 (1-(4-pyridinyl)-3-(2-quinolinyl)-2-propen-1-one) or epigallocatechin-3-gallate (EGCG) (298, 299) are known to considerably suppress tumor cell proliferation (Table 1). Some other anticancer agents like curcumin are also known to stall cancer progression by suppressing the PFK, hexokinase, Glut-4 expression both at mRNA and protein levels (300) (Table 1). Inhibition of PFKFB3 with pharmacological inhibitors like 3-(3-pyridinyl)-1-(4-pyridinyl)-2-propen-1-one (3-PO) in endothelial cells also leads to the suppression of the enhanced glycolysis (298, 301) (Table 1). Though the detailed molecular mechanism is unknown, it is assumed that this leads to improved tumor vasculatures through better adhesion of the pericytes (301). Blocking glycolysis in endothelial cells is thus also emerging as a novel therapeutic approach in cancer therapy. Pharmacological inhibitors of hexokinase and PFKFB3 like 2-DG and PFK15 have been successful in causing cytotoxicity in endothelial cells showing promise as therapeutic agents for cancer (119) (Table 1). Another metabolic analog that interferes with glycolysis and thereby tumor progression is 3-bromopyruvate (3-BP). 3-BP is known to suppress the expression of lactate transporter, MCT1 (302) as well as interfere with the activity of hexokinase (303) (Table 1). Taken together, in cases of multiple myeloma, treatment with 3-BP reduced the ATP level to 10% of untreated cells within 1 h leading to cytotoxicity (302). In addition to 3-BP, AZD3965, an inhibitor of MCT 1/2 targeting the transfer of lactate between cancer and cancer/stromal cells (Table 1) has been developed and is being tested for clinical efficacy (304). Another hexokinase and MCT1 inhibitor, Lonidamine has shown promising selective anticancer effects and has reached phase II of clinical trials (Table 1) (305–307). Similarly, caulerpin, a secondary metabolite, is presently being speculated for its anticancer property as its long-term application was found to interfere with the glycolytic machinery through AMPK pathway (308) (Table 1).

Although the conclusive picture of the signaling cascade that regulate the molecular remodeling in CAFs is yet to emerge, it will be interesting to speculate the candidature of molecules like TGF-β and c-Myc as potential drug targets (Table 1). TGF-β reportedly suppresses the TCA cycle enzyme isocitrate dehydrogenase (237) through a TGF-β/PDGF-mediated pathway thereby promoting glycolytic metabolism in CAF. Similarly, high activation of c-Myc promotes the expression of LDH-A and Glut-1 that are essential in maintaining the glycolytic flux (239).

#### Targeting Amino Acid Metabolism

The catabolism of l-arginine and tryptophan plays a significant role in tumor progression and immunity. Enhanced intra-tumoral RNS production due to increased metabolism of arginine in the TME induces CCL2 chemokine nitration and hinders T cell infiltration (309). It was reported that preconditioning of the TME with novel drugs that inhibit CCL2 modification facilitates CTL invasion of the tumor, suggesting their effectiveness in cancer immunotherapy (309).

Exhausted and antigen-tolerant T cells might be reactivated using IDO inhibitors resulting in increased tryptophan levels. This may be more beneficial in cancer therapy than increasing glycolysis as differential effects on immune cells could be obtained since glycolytic metabolic pathways are used both by cancer and T cells for their growth and survival. Two of the IDO inhibitors 1-methyl-tryptophan (1-MT) (310) and INCB024360 (311) have shown antitumor activity in mice tumor models due to increased T cell proliferation (Table 1). Downregulation of IDO has been observed with imatinib, a Bcr-Abl tyrosine kinase inhibitor in gastrointestinal tumors that resulted in the activation of CD8^+^ T cells and induced Treg cell apoptosis leading to enhanced antitumor effects (312) (Table 1). Imatinib could also inhibit Lck-mediated TCR signaling (313, 314) that is important for maximum glucose uptake through Glut-1 (86). This may lead to negative effects on T cell transition and therefore detrimental effects on antitumor immune responses.

#### Targeting Lipid Metabolism

Unlike T effector cells, Tregs depend on lipid metabolism for their differentiation and this provides an opportunity to differentially target these cells by using lipid oxidation blockers. An important role of FAO key enzyme, carnitine palmitoyl transferase 1a (CPT-1a) has been demonstrated in cancer cell survival in conditions of energy stress as it rewires the cancer cell metabolism (315, 316). Treatment of Tregs with etomoxir, a CPT-1a inhibitor resulted in differential suppression of Treg generation but not Th1 cells (101) making etomoxir a promising metabolic modulator for cancer therapy (Table 1). 3-hydroxy-3-methylglutaryl-CoA reductase (HMGCR), a rate-limiting enzyme for the synthesis of cholesterol and isoprenoid lipids has been targeted using a general lipid-synthesis inhibitor, 25-hydroxycholesterol, and drugs such as simvastatin, atorvastatin, and lovastatin to impair the activity of Tregs as HMGCR is needed for proliferation of Tregs (317) (Table 1).

As CAAs provide a source of energy to cancer cells in the form of fatty acids, preventing induction of CAA phenotype provides another promising therapeutic strategy. Several thiazolidinediones (troglitazone, rosiglitazone, and pioglitazone) that are ligands for PPARγ, which regulates the terminal differentiation of adipocytes (318), have been shown to inhibit the dedifferentiation of adipocytes to CAA (319) (Table 1). However, some of the thiazolidinediones are associated with cardiovascular side effects (120) and hence strategies that block cancer cells from using energy supplied by the CAAs have been developed using FABP4 inhibitors (245) and metformin (Table 1). Interestingly, metformin plays dual role in cancer therapy by inhibiting both the use of CAA-supplied energy by cancer cells as well as cancer cell-induced metabolic changes in the CAFs. Metformin has been found to block adipocyte-mediated lipid accumulation in ovarian cancer cells (320) and reverses the CAF phenotype induced by cancer cells by restoring caveolin expression in the fibroblasts (321).

### Targeting Acidic TME

The acidic TME that alters tumor metabolism has been targeted with systemic buffer therapy using buffers such as lysine, sodium bicarbonate, or 2-imidazole-1-yl-3-ethoxycarbonylpropionic acid (IEPA) (Table 1) to alkalize the TME leading to reduced tumor growth and metastasis (322–324) and to increase the activity of some drugs that otherwise remain inactive in acidic environments (325, 326). Further, proton pump inhibition to manipulate tumor pH and increase the intracellular acidity has also been employed as a therapeutic strategy. Bafilomycin A1 (327) and archazolid (328), V-ATPase proton pump inhibitors showed anticancer activity in several types of cancers. Therapeutic effects of several other proton pump inhibitors such as omeprazole, esomeprazole, rabeprazole, pantoprazole, and lansoprazole (Table 1) have been investigated suggesting the potential of these inhibitors in cancer therapy (329). Another attractive target of acidic cancer cells is CAIX that is overexpressed in these cells due to extracellular acidosis (330). Several inhibitors such as sulfonamides, sulfamates, and sulfamides (Table 1) have been developed that bind to the catalytic site of the enzyme (331). *In vivo* efficacy of these compounds is currently under investigation; however, a significant reduction in tumor growth and metastasis has been observed in a mammary tumor model in mice with novel CAIX inhibitors (332). Since acidic TME modulates the activation of proton-sensing G-protein-coupled receptors (333), an agonist of GPR68 (Table 1) has been investigated and has shown inhibitory effects in malignant astrocyte proliferation. However, further understanding of the molecular signaling and mechanisms of how these receptors alter tumor metabolism is essential to develop novel small molecules for cancer therapy.

## Conclusion and Future Direction

Although insight into the intricate nature of metabolic cooperation between the tumor cells and various host cells that it interacts within the microenvironment are emerging at the present, their potential as therapeutic targets is already indicated by the encouraging results from the studies with modifiers of lactate transport (MCT1) (17, 262). More recent studies showing the immune suppressive potential of lactic acid (3) emphasizes the importance of this metabolite that has an important role in the metabolic crosstalk between cancer cells and fibroblasts as well as the immune cells. Similarly, the dependence of cancer cells on the CAF for glutamine uptake (334) as well as support provided by the endothelial cells for their growth (119) highlights the importance of metabolic cooperation that can be used as a target for developing therapies (72). Further, the revelations on the contributions of immune modulation by the glycolytic inhibitor 2-DG to radiosensitization of tumors (258, 259) and its potential to impair the tumorigenesis (335) lend support to the proposition of targeting host–tumor interactions by metabolic modifiers for enhancing therapeutic gain. Furthermore, development of therapies that enhance the responses mediated by effector and memory T cells while reducing the suppressive functions of Tregs hold significant potential for cancer immunotherapy. Several therapeutic strategies for regulating Treg cell metabolism have been developed [reviewed in Ref. (336)]. Indeed, many of the currently employed therapeutic modalities target the metabolic pathways or the signaling cascades that govern them (Figures 1 and 2; Table 1). However, the metabolic cooperation as well as competition that set the metabolic fitness of different types of cells present in the TME needs further investigations to achieve better clinical outcomes. Therefore, while using engineered T cells during ACT or chimeric antigen receptor (CAR) T cell therapies, it is important to consider that limiting nutrients and other conditions in the TME will influence the effectiveness of these strategies. In addition, the cells of the innate immune system may recognize signals released from the cancer cells thereby supporting carcinogenesis. Pattern recognition receptors (PRRs) present on the surfaces of macrophages and other cells recognize different types of obnoxious stimuli present in their immediate vicinity and activate intracellular signaling cascades, which generally leads to the induction of pro-inflammatory response through upregulation of several genes (337, 338). There are several families of PRRs; however, the best characterized are the TLRs and the NOD-like receptors (NLRs). The ability of damage-associated molecular patterns (DAMP) released from dying cells (apoptotic and necrotic) has been widely implicated in tumorigenesis beyond pathogen-driven neoplasms (338) and may facilitate the interaction of tumor cells and cells of the immune network. Understanding the nature of metabolic reprograming in PRR-mediated tumor progression is required for developing therapeutic strategies that specifically target this aspect of the TME. Although anticancer therapies targeting the metabolic reprogramming have not been completely developed so far, increasing knowledge about this phenotype coupled with the insights gained about the TME is expected to result in the development of novel anticancer therapies in the near future.

## Author Contributions

All three authors jointly developed the structure and arguments for the paper, prepared the manuscript, reviewed, and approved the final manuscript.

## Conflict of Interest Statement

The authors declare that the research was conducted in the absence of any commercial or financial relationships that could be construed as a potential conflict of interest.

## References

[1] Warburg O The Metabolism of Tumors. London: Constable and Co. (1930).

[2] Martinez-Outschoorn U Sotgia F Lisanti MP. Tumor microenvironment and metabolic synergy in breast cancers: critical importance of mitochondrial fuels and function. Semin Oncol (2014) 41:195–216. 10.1053/j.seminoncol.2014.03.002 24787293

[3] Choi SY Collins CC Gout PW Wang Y. Cancer-generated lactic acid: a regulatory, immunosuppressive metabolite? J Pathol (2013) 230:350–5. 10.1002/path.4218 23729358 PMC3757307

[4] Pearce EL Pearce EJ. Metabolic pathways in immune cell activation and quiescence. Immunity (2013) 38:633–43. 10.1016/j.immuni.2013.04.005 23601682 PMC3654249

[5] Buck MD O’Sullivan D Pearce EL. T cell metabolism drives immunity. J Exp Med (2015) 212:1345–60. 10.1084/jem.20151159 26261266 PMC4548052

[6] O’Sullivan D Pearce EL. Targeting T cell metabolism for therapy. Trends Immunol (2015) 36:71–80. 10.1016/j.it.2014.12.004 25601541 PMC4323623

[7] O’Sullivan D van der Windt GJ Huang SC Curtis JD Chang CH Buck MD Memory CD8(+) T cells use cell-intrinsic lipolysis to support the metabolic programming necessary for development. Immunity (2014) 41:75–88. 10.1016/j.immuni.2014.06.005 25001241 PMC4120664

[8] Lunt SJ Chaudary N Hill RP. The tumor microenvironment and metastatic disease. Clin Exp Metastasis (2009) 26:19–34. 10.1007/s10585-008-9182-2 18543068

[9] Cairns RA Harris IS Mak TW. Regulation of cancer cell metabolism. Nat Rev Cancer (2011) 11:85–95. 10.1038/nrc2981 21258394

[10] Pavlides S Vera I Gandara R Sneddon S Pestell RG Mercier I Warburg meets autophagy: cancer-associated fibroblasts accelerate tumor growth and metastasis via oxidative stress, mitophagy, and aerobic glycolysis. Antioxid Redox Signal (2012) 16:1264–84. 10.1089/ars.2011.4243 21883043 PMC3324816

[11] Eckert AW Wickenhauser C Salins PC Kappler M Bukur J Seliger B. Clinical relevance of the tumor microenvironment and immune escape of oral squamous cell carcinoma. J Transl Med (2016) 14:85. 10.1186/s12967-016-0828-6 27044404 PMC4820994

[12] Arora A Singh S Bhatt AN Pandey S Sandhir R Dwarakanath BS Interplay between metabolism and oncogenic process: role of microRNAs. Transl Oncogenomics (2015) 7:11–27. 10.4137/TOG.S29652 26740741 PMC4696840

[13] Kouidhi S Noman MZ Kieda C Elgaaied AB Chouaib S. Intrinsic and tumor microenvironment-induced metabolism adaptations of T cells and impact on their differentiation and function. Front Immunol (2016) 7:114. 10.3389/fimmu.2016.00114 27066006 PMC4810024

[14] Wu W Zhao S. Metabolic changes in cancer: beyond the Warburg effect. Acta Biochim Biophys Sin (Shanghai) (2013) 45:18–26. 10.1093/abbs/gms104 23257292

[15] Wang T Liu G Wang R. The intercellular metabolic interplay between tumor and immune cells. Front Immunol (2014) 5:358. 10.3389/fimmu.2014.00358 25120544 PMC4112791

[16] Siska PJ Rathmell JC. T cell metabolic fitness in antitumor immunity. Trends Immunol (2015) 36:257–64. 10.1016/j.it.2015.02.007 25773310 PMC4393792

[17] Pisarsky L Bill R Fagiani E Dimeloe S Goosen RW Hagmann J Targeting metabolic symbiosis to overcome resistance to anti-angiogenic therapy. Cell Rep (2016) 15:1161–74. 10.1016/j.celrep.2016.04.028 27134168 PMC4870473

[18] Nakajima EC Van Houten B. Metabolic symbiosis in cancer: refocusing the Warburg lens. Mol Carcinog (2013) 52:329–37. 10.1002/mc.21863 22228080 PMC9972501

[19] Molon B Cali B Viola A. T cells and cancer: how metabolism shapes immunity. Front Immunol (2016) 7:20. 10.3389/fimmu.2016.00020 26870036 PMC4740780

[20] Mockler MB Conroy MJ Lysaght J. Targeting T cell immunometabolism for cancer immunotherapy; understanding the impact of the tumor microenvironment. Front Oncol (2014) 4:107. 10.3389/fonc.2014.00107 24904823 PMC4032940

[21] Kalluri R Zeisberg M Fibroblasts in cancer. Nat Rev Cancer (2006) 6:392–401. 10.1038/nrc1877 16572188

[22] Pietras K Ostman A. Hallmarks of cancer: interactions with the tumor stroma. Exp Cell Res (2010) 316:1324–31. 10.1016/j.yexcr.2010.02.045 20211171

[23] Amatangelo MD Bassi DE Klein-Szanto AJ Cukierman E. Stroma-derived three-dimensional matrices are necessary and sufficient to promote desmoplastic differentiation of normal fibroblasts. Am J Pathol (2005) 167:475–88. 10.1016/S0002-9440(10)62991-4 16049333 PMC1603576

[24] Siemann DW. The unique characteristics of tumor vasculature and preclinical evidence for its selective disruption by tumor-vascular disrupting agents. Cancer Treat Rev (2011) 37:63–74. 10.1016/j.ctrv.2010.05.001 20570444 PMC2958232

[25] Nagy JA Chang SH Dvorak AM Dvorak HF. Why are tumour blood vessels abnormal and why is it important to know? Br J Cancer (2009) 100:865–9. 10.1038/sj.bjc.6604929 19240721 PMC2661770

[26] Chiche J Brahimi-Horn MC Pouyssegur J. Tumour hypoxia induces a metabolic shift causing acidosis: a common feature in cancer. J Cell Mol Med (2010) 14:771–94. 10.1111/j.1582-4934.2009.00994.x 20015196 PMC3823111

[27] Peppicelli S Andreucci E Ruzzolini J Margheri F Laurenzana A Bianchini F Acidity of microenvironment as a further driver of tumor metabolic reprogramming. J Clin Cell Immunol (2017) 8:485–9. 10.4172/2155-9899.1000485

[28] Justus CR Sanderlin EJ Yang LV. Molecular connections between cancer cell metabolism and the tumor microenvironment. Int J Mol Sci (2015) 16:11055–86. 10.3390/ijms160511055 25988385 PMC4463690

[29] Chen JL Lucas JE Schroeder T Mori S Wu J Nevins J The genomic analysis of lactic acidosis and acidosis response in human cancers. PLoS Genet (2008) 4:e1000293. 10.1371/journal.pgen.1000293 19057672 PMC2585811

[30] Xie J Wu H Dai C Pan Q Ding Z Hu D Beyond Warburg effect – dual metabolic nature of cancer cells. Sci Rep (2014) 4:4927 10.1038/srep04927 24820099 PMC4018627

[31] DeBerardinis RJ Lum JJ Hatzivassiliou G Thompson CB. The biology of cancer: metabolic reprogramming fuels cell growth and proliferation. Cell Metab (2008) 7:11–20. 10.1016/j.cmet.2007.10.002 18177721

[32] Eigenbrodt E Reinacher M Scheefers-Borchel U Scheefers H Friis R. Double role for pyruvate kinase type M2 in the expansion of phosphometabolite pools found in tumor cells. Crit Rev Oncog (1992) 3:91–115. 1532331

[33] Warburg O On the origin of cancer cells. Science (1956) 123:309–14. 10.1126/science.123.3191.309 13298683

[34] Bost F Decoux-Poullot AG Tanti JF Clavel S. Energy disruptors: rising stars in anticancer therapy? Oncogenesis (2016) 5:e188. 10.1038/oncsis.2015.46 26779810 PMC4728676

[35] Slaninova V Krafcikova M Perez-Gomez R Steffal P Trantirek L Bray SJ Notch stimulates growth by direct regulation of genes involved in the control of glycolysis and the tricarboxylic acid cycle. Open Biol (2016) 6:150155. 10.1098/rsob.150155 26887408 PMC4772804

[36] Dang CV O’Donnell KA Zeller KI Nguyen T Osthus RC Li F. The c-Myc target gene network. Semin Cancer Biol (2006) 16:253–64. 10.1016/j.semcancer.2006.07.014 16904903

[37] Wilde BR Ayer DE. Interactions between Myc and MondoA transcription factors in metabolism and tumourigenesis. Br J Cancer (2015) 113:1529–33. 10.1038/bjc.2015.360 26469830 PMC4705882

[38] Yeung SJ Pan J Lee MH Roles of p53, MYC and HIF-1 in regulating glycolysis – the seventh hallmark of cancer. Cell Mol Life Sci (2008) 65:3981–99. 10.1007/s00018-008-8224-x 18766298 PMC11131737

[39] Ke Q Costa M. Hypoxia-inducible factor-1 (HIF-1). Mol Pharmacol (2006) 70:1469–80. 10.1124/mol.106.027029 16887934

[40] Papandreou I Cairns RA Fontana L Lim AL Denko NC. HIF-1 mediates adaptation to hypoxia by actively downregulating mitochondrial oxygen consumption. Cell Metab (2006) 3:187–97. 10.1016/j.cmet.2006.01.012 16517406

[41] Kim JW Tchernyshyov I Semenza GL Dang CV. HIF-1-mediated expression of pyruvate dehydrogenase kinase: a metabolic switch required for cellular adaptation to hypoxia. Cell Metab (2006) 3:177–85. 10.1016/j.cmet.2006.02.002 16517405

[42] Semenza GL. Regulation of cancer cell metabolism by hypoxia-inducible factor 1. Semin Cancer Biol (2009) 19:12–6. 10.1016/j.semcancer.2008.11.009 19114105

[43] Swietach P Hulikova A Vaughan-Jones RD Harris AL. New insights into the physiological role of carbonic anhydrase IX in tumour pH regulation. Oncogene (2010) 29:6509–21. 10.1038/onc.2010.455 20890298

[44] Zhang H Bosch-Marce M Shimoda LA Tan YS Baek JH Wesley JB Mitochondrial autophagy is an HIF-1-dependent adaptive metabolic response to hypoxia. J Biol Chem (2008) 283:10892–903. 10.1074/jbc.M800102200 18281291 PMC2447655

[45] Fukuda R Zhang H Kim JW Shimoda L Dang CV Semenza GL. HIF-1 regulates cytochrome oxidase subunits to optimize efficiency of respiration in hypoxic cells. Cell (2007) 129:111–22. 10.1016/j.cell.2007.01.047 17418790

[46] Guertin DA Sabatini DM Defining the role of mTOR in cancer. Cancer Cell (2007) 12:9–22. 10.1016/j.ccr.2007.05.008 17613433

[47] Browne GJ Proud CG. A novel mTOR-regulated phosphorylation site in elongation factor 2 kinase modulates the activity of the kinase and its binding to calmodulin. Mol Cell Biol (2004) 24:2986–97. 10.1128/MCB.24.7.2986-2997.2004 15024086 PMC371112

[48] Vaupel P Kallinowski F Okunieff P. Blood flow, oxygen and nutrient supply, and metabolic microenvironment of human tumors: a review. Cancer Res (1989) 49:6449–65. 2684393

[49] Vaupel P. Tumor microenvironmental physiology and its implications for radiation oncology. Semin Radiat Oncol (2004) 14:198–206. 10.1016/j.semradonc.2004.04.008 15254862

[50] Helmlinger G Sckell A Dellian M Forbes NS Jain RK. Acid production in glycolysis-impaired tumors provides new insights into tumor metabolism. Clin Cancer Res (2002) 8:1284–91. 11948144

[51] Williams AC Collard TJ Paraskeva C. An acidic environment leads to p53 dependent induction of apoptosis in human adenoma and carcinoma cell lines: implications for clonal selection during colorectal carcinogenesis. Oncogene (1999) 18:3199–204. 10.1038/sj.onc.1202660 10359525

[52] Putney LK Barber DL. Na-H exchange-dependent increase in intracellular pH times G2/M entry and transition. J Biol Chem (2003) 278:44645–9. 10.1074/jbc.M308099200 12947095

[53] Morita T. Low pH leads to sister-chromatid exchanges and chromosomal aberrations, and its clastogenicity is S-dependent. Mutat Res (1995) 334:301–8. 10.1016/0165-1161(95)90067-5 7753094

[54] Morita T Nagaki T Fukuda I Okumura K. Clastogenicity of low pH to various cultured mammalian cells. Mutat Res (1992) 268:297–305. 10.1016/0027-5107(92)90235-T 1379335

[55] Snabaitis AK Cuello F Avkiran M. Protein kinase B/Akt phosphorylates and inhibits the cardiac Na+/H+ exchanger NHE1. Circ Res (2008) 103:881–90. 10.1161/CIRCRESAHA.108.175877 18757828

[56] Meima ME Webb BA Witkowska HE Barber DL. The sodium-hydrogen exchanger NHE1 is an Akt substrate necessary for actin filament reorganization by growth factors. J Biol Chem (2009) 284:26666–75. 10.1074/jbc.M109.019448 19622752 PMC2785354

[57] Jensen DH Therkildsen MH Dabelsteen E. A reverse Warburg metabolism in oral squamous cell carcinoma is not dependent upon myofibroblasts. J Oral Pathol Med (2015) 44:714–21. 10.1111/jop.12297 25420473

[58] Starska K Forma E Jozwiak P Brys M Lewy-Trenda I Brzezinska-Blaszczyk E Gene and protein expression of glucose transporter 1 and glucose transporter 3 in human laryngeal cancer-the relationship with regulatory hypoxia-inducible factor-1alpha expression, tumor invasiveness, and patient prognosis. Tumour Biol (2015) 36:2309–21. 10.1007/s13277-014-2838-4 25412955 PMC4428538

[59] Eckert AW Lautner MH Schutze A Taubert H Schubert J Bilkenroth U Coexpression of hypoxia-inducible factor-1alpha and glucose transporter-1 is associated with poor prognosis in oral squamous cell carcinoma patients. Histopathology (2011) 58:1136–47. 10.1111/j.1365-2559.2011.03806.x 21438910

[60] Dwarakanath B Jain V Targeting glucose metabolism with 2-deoxy-D-glucose for improving cancer therapy. Future Oncol (2009) 5:581–5. 10.2217/fon.09.44 19519197

[61] Gambhir SS. Molecular imaging of cancer with positron emission tomography. Nat Rev Cancer (2002) 2:683–93. 10.1038/nrc882 12209157

[62] Kim JW Dang CV. Cancer’s molecular sweet tooth and the Warburg effect. Cancer Res (2006) 66:8927–30. 10.1158/0008-5472.CAN-06-1501 16982728

[63] Cao X Fang L Gibbs S Huang Y Dai Z Wen P Glucose uptake inhibitor sensitizes cancer cells to daunorubicin and overcomes drug resistance in hypoxia. Cancer Chemother Pharmacol (2007) 59:495–505. 10.1007/s00280-006-0291-9 16906425

[64] Xu RH Pelicano H Zhou Y Carew JS Feng L Bhalla KN Inhibition of glycolysis in cancer cells: a novel strategy to overcome drug resistance associated with mitochondrial respiratory defect and hypoxia. Cancer Res (2005) 65:613–21. 15695406

[65] Bhatt AN Chauhan A Khanna S Rai Y Singh S Soni R Transient elevation of glycolysis confers radio-resistance by facilitating DNA repair in cells. BMC Cancer (2015) 15:335. 10.1186/s12885-015-1368-9 25925410 PMC4425929

[66] Kuo W Lin J Tang TK. Human glucose-6-phosphate dehydrogenase (G6PD) gene transforms NIH 3T3 cells and induces tumors in nude mice. Int J Cancer (2000) 85:857–64. 10.1002/(SICI)1097-0215(20000315)85:6<857::AID-IJC20>3.3.CO;2-L 10709108

[67] Ramos-Montoya A Lee WN Bassilian S Lim S Trebukhina RV Kazhyna MV Pentose phosphate cycle oxidative and nonoxidative balance: a new vulnerable target for overcoming drug resistance in cancer. Int J Cancer (2006) 119:2733–41. 10.1002/ijc.22227 17019714

[68] Zanuy M Ramos-Montoya A Villacanas O Canela N Miranda A Aguilar E Cyclin-dependent kinases 4 and 6 control tumor progression and direct glucose oxidation in the pentose cycle. Metabolomics (2012) 8:454–64. 10.1007/s11306-011-0328-x 22661920 PMC3361763

[69] Beloribi-Djefaflia S Vasseur S Guillaumond F. Lipid metabolic reprogramming in cancer cells. Oncogenesis (2016) 5:e189. 10.1038/oncsis.2015.49 26807644 PMC4728678

[70] Santos CR Schulze A Lipid metabolism in cancer. FEBS J (2012) 279:2610–23. 10.1111/j.1742-4658.2012.08644.x 22621751

[71] Hensley CT Wasti AT DeBerardinis RJ. Glutamine and cancer: cell biology, physiology, and clinical opportunities. J Clin Invest (2013) 123:3678–84. 10.1172/JCI69600 23999442 PMC3754270

[72] Tajan M Vousden KH. The Quid Pro Quo of the tumor/stromal interaction. Cell Metab (2016) 24:645–6. 10.1016/j.cmet.2016.10.017 27829131

[73] Wang Q Hardie RA Hoy AJ van Geldermalsen M Gao D Fazli L Targeting ASCT2-mediated glutamine uptake blocks prostate cancer growth and tumour development. J Pathol (2015) 236:278–89. 10.1002/path.4518 25693838 PMC4973854

[74] van Geldermalsen M Wang Q Nagarajah R Marshall AD Thoeng A Gao D ASCT2/SLC1A5 controls glutamine uptake and tumour growth in triple-negative basal-like breast cancer. Oncogene (2016) 35:3201–8. 10.1038/onc.2015.381 26455325 PMC4914826

[75] Lamonte G Tang X Chen JL Wu J Ding CK Keenan MM Acidosis induces reprogramming of cellular metabolism to mitigate oxidative stress. Cancer Metab (2013) 1:23. 10.1186/2049-3002-1-23 24359630 PMC4178214

[76] Perez-Escuredo J Dadhich RK Dhup S Cacace A Van Hee VF De Saedeleer CJ Lactate promotes glutamine uptake and metabolism in oxidative cancer cells. Cell Cycle (2016) 15:72–83. 10.1080/15384101.2015.1120930 26636483 PMC4825768

[77] Ananieva E. Targeting amino acid metabolism in cancer growth and anti-tumor immune response. World J Biol Chem (2015) 6:281–9. 10.4331/wjbc.v6.i4.281 26629311 PMC4657121

[78] Pearce EL Poffenberger MC Chang CH Jones RG. Fueling immunity: insights into metabolism and lymphocyte function. Science (2013) 342:1242454. 10.1126/science.1242454 24115444 PMC4486656

[79] Greiner EF Guppy M Brand K. Glucose is essential for proliferation and the glycolytic enzyme induction that provokes a transition to glycolytic energy production. J Biol Chem (1994) 269:31484–90. 7989314

[80] Carr EL Kelman A Wu GS Gopaul R Senkevitch E Aghvanyan A Glutamine uptake and metabolism are coordinately regulated by ERK/MAPK during T lymphocyte activation. J Immunol (2010) 185:1037–44. 10.4049/jimmunol.0903586 20554958 PMC2897897

[81] Vesely MD Schreiber RD. Cancer immunoediting: antigens, mechanisms, and implications to cancer immunotherapy. Ann N Y Acad Sci (2013) 1284:1–5. 10.1111/nyas.12105 23651186 PMC3648872

[82] Chang CH Qiu J O’Sullivan D Buck MD Noguchi T Curtis JD Metabolic competition in the tumor microenvironment is a driver of cancer progression. Cell (2015) 162:1229–41. 10.1016/j.cell.2015.08.016 26321679 PMC4864363

[83] Crespo J Sun H Welling TH Tian Z Zou W. T cell anergy, exhaustion, senescence, and stemness in the tumor microenvironment. Curr Opin Immunol (2013) 25:214–21. 10.1016/j.coi.2012.12.003 23298609 PMC3636159

[84] Guppy M Greiner E Brand K. The role of the Crabtree effect and an endogenous fuel in the energy metabolism of resting and proliferating thymocytes. Eur J Biochem (1993) 212:95–9. 10.1111/j.1432-1033.1993.tb17637.x 8444168

[85] Marelli-Berg FM Fu H Mauro C. Molecular mechanisms of metabolic reprogramming in proliferating cells: implications for T-cell-mediated immunity. Immunology (2012) 136:363–9. 10.1111/j.1365-2567.2012.03583.x 22384794 PMC3401974

[86] Jacobs SR Herman CE Maciver NJ Wofford JA Wieman HL Hammen JJ Glucose uptake is limiting in T cell activation and requires CD28-mediated Akt-dependent and independent pathways. J Immunol (2008) 180:4476–86. 10.4049/jimmunol.180.7.4476 18354169 PMC2593791

[87] Fox CJ Hammerman PS Thompson CB. Fuel feeds function: energy metabolism and the T-cell response. Nat Rev Immunol (2005) 5:844–52. 10.1038/nri1710 16239903

[88] Bradley LM Haynes L Swain SL. IL-7: maintaining T-cell memory and achieving homeostasis. Trends Immunol (2005) 26:172–6. 10.1016/j.it.2005.01.004 15745860

[89] Hammerman PS Fox CJ Thompson CB. Beginnings of a signal-transduction pathway for bioenergetic control of cell survival. Trends Biochem Sci (2004) 29:586–92. 10.1016/j.tibs.2004.09.008 15501677

[90] Plas DR Rathmell JC Thompson CB. Homeostatic control of lymphocyte survival: potential origins and implications. Nat Immunol (2002) 3:515–21. 10.1038/ni0602-515 12032565

[91] Nakaya M Xiao Y Zhou X Chang JH Chang M Cheng X Inflammatory T cell responses rely on amino acid transporter ASCT2 facilitation of glutamine uptake and mTORC1 kinase activation. Immunity (2014) 40:692–705. 10.1016/j.immuni.2014.04.007 24792914 PMC4074507

[92] Rodriguez PC Zea AH Culotta KS Zabaleta J Ochoa JB Ochoa AC. Regulation of T cell receptor CD3zeta chain expression by L-arginine. J Biol Chem (2002) 277:21123–9. 10.1074/jbc.M110675200 11950832

[93] Rodriguez PC Quiceno DG Ochoa AC. L-arginine availability regulates T-lymphocyte cell-cycle progression. Blood (2007) 109:1568–73. 10.1182/blood-2006-06-031856 17023580 PMC1794048

[94] Chen L Flies DB. Molecular mechanisms of T cell co-stimulation and co-inhibition. Nat Rev Immunol (2013) 13:227–42. 10.1038/nri3405 23470321 PMC3786574

[95] Frauwirth KA Thompson CB Regulation of T lymphocyte metabolism. J Immunol (2004) 172:4661–5. 10.4049/jimmunol.172.8.4661 15067038

[96] Dan HC Ebbs A Pasparakis M Van Dyke T Basseres DS Baldwin AS Akt-dependent activation of mTORC1 complex involves phosphorylation of mTOR (mammalian target of rapamycin) by IkappaB kinase alpha (IKKalpha). J Biol Chem (2014) 289:25227–40. 10.1074/jbc.M114.554881 24990947 PMC4155685

[97] Morita M Gravel SP Hulea L Larsson O Pollak M St-Pierre J mTOR coordinates protein synthesis, mitochondrial activity and proliferation. Cell Cycle (2015) 14:473–80. 10.4161/15384101.2014.991572 25590164 PMC4615141

[98] Wang R Dillon CP Shi LZ Milasta S Carter R Finkelstein D The transcription factor Myc controls metabolic reprogramming upon T lymphocyte activation. Immunity (2011) 35:871–82. 10.1016/j.immuni.2011.09.021 22195744 PMC3248798

[99] Peter C Waldmann H Cobbold SP. mTOR signalling and metabolic regulation of T cell differentiation. Curr Opin Immunol (2010) 22:655–61. 10.1016/j.coi.2010.08.010 20833524

[100] Powell JD Delgoffe GM. The mammalian target of rapamycin: linking T cell differentiation, function, and metabolism. Immunity (2010) 33:301–11. 10.1016/j.immuni.2010.09.002 20870173 PMC2962404

[101] Michalek RD Gerriets VA Jacobs SR Macintyre AN MacIver NJ Mason EF Cutting edge: distinct glycolytic and lipid oxidative metabolic programs are essential for effector and regulatory CD4+ T cell subsets. J Immunol (2011) 186:3299–303. 10.4049/jimmunol.1003613 21317389 PMC3198034

[102] Rubtsov YP Niec RE Josefowicz S Li L Darce J Mathis D Stability of the regulatory T cell lineage in vivo. Science (2010) 329:1667–71. 10.1126/science.1191996 20929851 PMC4262151

[103] van der Windt GJ Everts B Chang CH Curtis JD Freitas TC Amiel E Mitochondrial respiratory capacity is a critical regulator of CD8+ T cell memory development. Immunity (2012) 36:68–78. 10.1016/j.immuni.2011.12.007 22206904 PMC3269311

[104] Song JJ Ott HC. Organ engineering based on decellularized matrix scaffolds. Trends Mol Med (2011) 17:424–32. 10.1016/j.molmed.2011.03.005 21514224

[105] Darby IA Laverdet B Bonte F Desmouliere A Fibroblasts and myofibroblasts in wound healing. Clin Cosmet Investig Dermatol (2014) 7:301–11. 10.2147/CCID.S50046 PMC4226391 25395868

[106] Witz IP. The tumor microenvironment: the making of a paradigm. Cancer Microenviron (2009) 2(Suppl 1):9–17. 10.1007/s12307-009-0025-8 19701697 PMC2756342

[107] Bhome R Bullock MD Al Saihati HA Goh RW Primrose JN Sayan AE A top-down view of the tumor microenvironment: structure, cells and signaling. Front Cell Dev Biol (2015) 3:33. 10.3389/fcell.2015.00033 26075202 PMC4448519

[108] Vannucci L. Stroma as an active player in the development of the tumor microenvironment. Cancer Microenviron (2015) 8:159–66. 10.1007/s12307-014-0150-x 25106539 PMC4715002

[109] Lee D Ham IH Son SY Han SU Kim YB Hur H. Intratumor stromal proportion predicts aggressive phenotype of gastric signet ring cell carcinomas. Gastric Cancer (2016) 1–11. 10.1007/s10120-016-0669-2 27858181

[110] Martinez-Outschoorn UE Lisanti MP Sotgia F. Catabolic cancer-associated fibroblasts transfer energy and biomass to anabolic cancer cells, fueling tumor growth. Semin Cancer Biol (2014) 25:47–60. 10.1016/j.semcancer.2014.01.005 24486645

[111] Fiaschi T Marini A Giannoni E Taddei ML Gandellini P De Donatis A Reciprocal metabolic reprogramming through lactate shuttle coordinately influences tumor-stroma interplay. Cancer Res (2012) 72:5130–40. 10.1158/0008-5472.CAN-12-1949 22850421

[112] Bonuccelli G Avnet S Grisendi G Salerno M Granchi D Dominici M Role of mesenchymal stem cells in osteosarcoma and metabolic reprogramming of tumor cells. Oncotarget (2014) 5:7575–88. 10.18632/oncotarget.2243 25277190 PMC4202145

[113] Whitaker-Menezes D Martinez-Outschoorn UE Lin Z Ertel A Flomenberg N Witkiewicz AK Evidence for a stromal-epithelial “lactate shuttle” in human tumors: MCT4 is a marker of oxidative stress in cancer-associated fibroblasts. Cell Cycle (2011) 10:1772–83. 10.4161/cc.10.11.15659 21558814 PMC3142461

[114] Shi H Jiang H Wang L Cao Y Liu P Xu X Overexpression of monocarboxylate anion transporter 1 and 4 in T24-induced cancer-associated fibroblasts regulates the progression of bladder cancer cells in a 3D microfluidic device. Cell Cycle (2015) 14:3058–65. 10.1080/15384101.2015.1053666 26125467 PMC4825595

[115] Colegio OR Lactic acid polarizes macrophages to a tumor-promoting state. Oncoimmunology (2016) 5:e1014774 10.1080/2162402X.2015.1014774 27141329 PMC4839384

[116] Colegio OR Chu NQ Szabo AL Chu T Rhebergen AM Jairam V Functional polarization of tumour-associated macrophages by tumour-derived lactic acid. Nature (2014) 513:559–63. 10.1038/nature13490 25043024 PMC4301845

[117] Gordon S Martinez FO. Alternative activation of macrophages: mechanism and functions. Immunity (2010) 32:593–604. 10.1016/j.immuni.2010.05.007 20510870

[118] Liu M Quek LE Sultani G Turner N. Epithelial-mesenchymal transition induction is associated with augmented glucose uptake and lactate production in pancreatic ductal adenocarcinoma. Cancer Metab (2016) 4:19. 10.1186/s40170-016-0160-x 27777765 PMC5066287

[119] Verdegem D Moens S Stapor P Carmeliet P. Endothelial cell metabolism: parallels and divergences with cancer cell metabolism. Cancer Metab (2014) 2:19. 10.1186/2049-3002-2-19 25250177 PMC4171726

[120] Romero IL Mukherjee A Kenny HA Litchfield LM Lengyel E. Molecular pathways: trafficking of metabolic resources in the tumor microenvironment. Clin Cancer Res (2015) 21:680–6. 10.1158/1078-0432.CCR-14-2198 25691772 PMC4333734

[121] Berg JM Tymoczko JL Stryer L Section 30.2, each organ has a unique metabolic profile. In: Freeman WH, editor. Biochemistry, 5th edn. New York: WH Freeman and Company (2002). Available from: https://www.ncbi.nlm.nih.gov/books/NBK22436/

[122] Hanahan D Coussens LM. Accessories to the crime: functions of cells recruited to the tumor microenvironment. Cancer Cell (2012) 21:309–22. 10.1016/j.ccr.2012.02.022 22439926

[123] Ho PC Bihuniak JD Macintyre AN Staron M Liu X Amezquita R Phosphoenolpyruvate is a metabolic checkpoint of anti-tumor T cell responses. Cell (2015) 162:1217–28. 10.1016/j.cell.2015.08.012 26321681 PMC4567953

[124] Li H Zhang J Chen SW Liu LL Li L Gao F Cancer-associated fibroblasts provide a suitable microenvironment for tumor development and progression in oral tongue squamous cancer. J Transl Med (2015) 13:198. 10.1186/s12967-015-0551-8 26094024 PMC4475624

[125] Izar B Joyce CE Goff S Cho NL Shah PM Sharma G Bidirectional cross talk between patient-derived melanoma and cancer-associated fibroblasts promotes invasion and proliferation. Pigment Cell Melanoma Res (2016) 29:656–68. 10.1111/pcmr.12513 27482935

[126] Olumi AF Grossfeld GD Hayward SW Carroll PR Tlsty TD Cunha GR. Carcinoma-associated fibroblasts direct tumor progression of initiated human prostatic epithelium. Cancer Res (1999) 59:5002–11. 10519415 10.1186/bcr138 PMC3300837

[127] LaGory EL Giaccia AJ. The ever-expanding role of HIF in tumour and stromal biology. Nat Cell Biol (2016) 18:356–65. 10.1038/ncb3330 27027486 PMC4898054

[128] Imtiyaz HZ Simon MC. Hypoxia-inducible factors as essential regulators of inflammation. Curr Top Microbiol Immunol (2010) 345:105–20. 10.1007/82_2010_74 20517715 PMC3144567

[129] Semenza GL. Targeting HIF-1 for cancer therapy. Nat Rev Cancer (2003) 3:721–32. 10.1038/nrc1187 13130303

[130] Bertout JA Patel SA Simon MC. The impact of O_2_ availability on human cancer. Nat Rev Cancer (2008) 8:967–75. 10.1038/nrc2540 18987634 PMC3140692

[131] Clambey ET McNamee EN Westrich JA Glover LE Campbell EL Jedlicka P Hypoxia-inducible factor-1 alpha-dependent induction of FoxP3 drives regulatory T-cell abundance and function during inflammatory hypoxia of the mucosa. Proc Natl Acad Sci U S A (2012) 109:E2784–93. 10.1073/pnas.1202366109 22988108 PMC3478644

[132] Dang EV Barbi J Yang HY Jinasena D Yu H Zheng Y Control of T(H)17/T(reg) balance by hypoxia-inducible factor 1. Cell (2011) 146:772–84. 10.1016/j.cell.2011.07.033 21871655 PMC3387678

[133] Ben-Shoshan J Maysel-Auslender S Mor A Keren G George J. Hypoxia controls CD4+CD25+ regulatory T-cell homeostasis via hypoxia-inducible factor-1alpha. Eur J Immunol (2008) 38:2412–8. 10.1002/eji.200838318 18792019

[134] Shi LZ Wang R Huang G Vogel P Neale G Green DR HIF1alpha-dependent glycolytic pathway orchestrates a metabolic checkpoint for the differentiation of TH17 and Treg cells. J Exp Med (2011) 208:1367–76. 10.1084/jem.20110278 21708926 PMC3135370

[135] Laoui D Van Overmeire E Di Conza G Aldeni C Keirsse J Morias Y Tumor hypoxia does not drive differentiation of tumor-associated macrophages but rather fine-tunes the M2-like macrophage population. Cancer Res (2014) 74:24–30. 10.1158/0008-5472.CAN-13-1196 24220244

[136] Stockmann C Doedens A Weidemann A Zhang N Takeda N Greenberg JI Deletion of vascular endothelial growth factor in myeloid cells accelerates tumorigenesis. Nature (2008) 456:814–8. 10.1038/nature07445 18997773 PMC3103772

[137] Wenes M Shang M Di Matteo M Goveia J Martin-Perez R Serneels J Macrophage metabolism controls tumor blood vessel morphogenesis and metastasis. Cell Metab (2016) 24:701–15. 10.1016/j.cmet.2016.09.008 27773694

[138] Galvan-Pena S O’Neill LA. Metabolic reprograming in macrophage polarization. Front Immunol (2014) 5:420. 10.3389/fimmu.2014.00420 25228902 PMC4151090

[139] Jetten N Verbruggen S Gijbels MJ Post MJ De Winther MP Donners MM. Anti-inflammatory M2, but not pro-inflammatory M1 macrophages promote angiogenesis in vivo. Angiogenesis (2014) 17:109–18. 10.1007/s10456-013-9381-6 24013945

[140] Jones CV Williams TM Walker KA Dickinson H Sakkal S Rumballe BA M2 macrophage polarisation is associated with alveolar formation during postnatal lung development. Respir Res (2013) 14:41. 10.1186/1465-9921-14-41 23560845 PMC3626876

[141] Balkwill FR Capasso M Hagemann T The tumor microenvironment at a glance. J Cell Sci (2012) 125:5591–6. 10.1242/jcs.116392 23420197

[142] Kang CW Dutta A Chang LY Mahalingam J Lin YC Chiang JM Apoptosis of tumor infiltrating effector TIM-3+CD8+ T cells in colon cancer. Sci Rep (2015) 5:15659. 10.1038/srep15659 26493689 PMC4616166

[143] Enninga EA Nevala WK Holtan SG Leontovich AA Markovic SN. Galectin-9 modulates immunity by promoting Th2/M2 differentiation and impacts survival in patients with metastatic melanoma. Melanoma Res (2016) 26:429–41. 10.1097/CMR.0000000000000281 27455380 PMC5929986

[144] Wu S Rhee KJ Albesiano E Rabizadeh S Wu X Yen HR A human colonic commensal promotes colon tumorigenesis via activation of T helper type 17 T cell responses. Nat Med (2009) 15:1016–22. 10.1038/nm.2015 19701202 PMC3034219

[145] Sharp SP Avram D Stain SC Lee EC. Local and systemic Th17 immune response associated with advanced stage colon cancer. J Surg Res (2017) 208:180–6. 10.1016/j.jss.2016.09.038 27993206 PMC6086724

[146] Han Y Ye A Bi L Wu J Yu K Zhang S. Th17 cells and interleukin-17 increase with poor prognosis in patients with acute myeloid leukemia. Cancer Sci (2014) 105:933–42. 10.1111/cas.12459 24890519 PMC4317867

[147] Cramer T Yamanishi Y Clausen BE Forster I Pawlinski R Mackman N HIF-1alpha is essential for myeloid cell-mediated inflammation. Cell (2003) 112:645–57. 10.1016/S0092-8674(03)00154-5 12628185 PMC4480774

[148] Corzo CA Condamine T Lu L Cotter MJ Youn JI Cheng P HIF-1alpha regulates function and differentiation of myeloid-derived suppressor cells in the tumor microenvironment. J Exp Med (2010) 207:2439–53. 10.1084/jem.20100587 20876310 PMC2964584

[149] Schroeder T Yuan H Viglianti BL Peltz C Asopa S Vujaskovic Z Spatial heterogeneity and oxygen dependence of glucose consumption in R3230Ac and fibrosarcomas of the Fischer 344 rat. Cancer Res (2005) 65:5163–71. 10.1158/0008-5472.CAN-04-3900 15958560

[150] MacIver NJ Michalek RD Rathmell JC. Metabolic regulation of T lymphocytes. Annu Rev Immunol (2013) 31:259–83. 10.1146/annurev-immunol-032712-095956 23298210 PMC3606674

[151] Cham CM Driessens G O’Keefe JP Gajewski TF. Glucose deprivation inhibits multiple key gene expression events and effector functions in CD8+ T cells. Eur J Immunol (2008) 38:2438–50. 10.1002/eji.200838289 18792400 PMC3008428

[152] Zheng Y Delgoffe GM Meyer CF Chan W Powell JD. Anergic T cells are metabolically anergic. J Immunol (2009) 183:6095–101. 10.4049/jimmunol.0803510 19841171 PMC2884282

[153] Chang CH Curtis JD Maggi LB Jr Faubert B Villarino AV O’Sullivan D Posttranscriptional control of T cell effector function by aerobic glycolysis. Cell (2013) 153:1239–51. 10.1016/j.cell.2013.05.016 23746840 PMC3804311

[154] Hanahan D Weinberg RA Hallmarks of cancer: the next generation. Cell (2011) 144:646–74. 10.1016/j.cell.2011.02.013 21376230

[155] Plas DR Thompson CB. Cell metabolism in the regulation of programmed cell death. Trends Endocrinol Metab (2002) 13:75–8. 10.1016/S1043-2760(01)00528-8 11854022

[156] Cory S Adams JM. The Bcl2 family: regulators of the cellular life-or-death switch. Nat Rev Cancer (2002) 2:647–56. 10.1038/nrc883 12209154

[157] Scharping NE Menk AV Moreci RS Whetstone RD Dadey RE Watkins SC The tumor microenvironment represses T cell mitochondrial biogenesis to drive intratumoral T cell metabolic insufficiency and dysfunction. Immunity (2016) 45:374–88. 10.1016/j.immuni.2016.07.009 27496732 PMC5207350

[158] Yang M Soga T Pollard PJ. Oncometabolites: linking altered metabolism with cancer. J Clin Invest (2013) 123:3652–8. 10.1172/JCI67228 23999438 PMC3754247

[159] Fischer K Hoffmann P Voelkl S Meidenbauer N Ammer J Edinger M Inhibitory effect of tumor cell-derived lactic acid on human T cells. Blood (2007) 109:3812–9. 10.1182/blood-2006-07-035972 17255361

[160] Fukumura D Xu L Chen Y Gohongi T Seed B Jain RK. Hypoxia and acidosis independently up-regulate vascular endothelial growth factor transcription in brain tumors in vivo. Cancer Res (2001) 61:6020–4. 11507045

[161] Calcinotto A Filipazzi P Grioni M Iero M De Milito A Ricupito A Modulation of microenvironment acidity reverses anergy in human and murine tumor-infiltrating T lymphocytes. Cancer Res (2012) 72:2746–56. 10.1158/0008-5472.CAN-11-1272 22593198

[162] Ohashi T Akazawa T Aoki M Kuze B Mizuta K Ito Y Dichloroacetate improves immune dysfunction caused by tumor-secreted lactic acid and increases antitumor immunoreactivity. Int J Cancer (2013) 133:1107–18. 10.1002/ijc.28114 23420584

[163] Sattler UG Meyer SS Quennet V Hoerner C Knoerzer H Fabian C Glycolytic metabolism and tumour response to fractionated irradiation. Radiother Oncol (2010) 94:102–9. 10.1016/j.radonc.2009.11.007 20036432

[164] Martinez D Vermeulen M Trevani A Ceballos A Sabatte J Gamberale R Extracellular acidosis induces neutrophil activation by a mechanism dependent on activation of phosphatidylinositol 3-kinase/Akt and ERK pathways. J Immunol (2006) 176:1163–71. 10.4049/jimmunol.176.2.1163 16394005

[165] Fischer B Muller B Fischer KG Baur N Kreutz W. Acidic pH inhibits non-MHC-restricted killer cell functions. Clin Immunol (2000) 96:252–63. 10.1006/clim.2000.4904 10964544

[166] Muller B Fischer B Kreutz W. An acidic microenvironment impairs the generation of non-major histocompatibility complex-restricted killer cells. Immunology (2000) 99:375–84. 10.1046/j.1365-2567.2000.00975.x 10712667 PMC2327168

[167] Tannahill GM Curtis AM Adamik J Palsson-McDermott EM McGettrick AF Goel G Succinate is an inflammatory signal that induces IL-1beta through HIF-1alpha. Nature (2013) 496:238–42. 10.1038/nature11986 23535595 PMC4031686

[168] Rubic T Lametschwandtner G Jost S Hinteregger S Kund J Carballido-Perrig N Triggering the succinate receptor GPR91 on dendritic cells enhances immunity. Nat Immunol (2008) 9:1261–9. 10.1038/ni.1657 18820681

[169] Villalba M Rathore MG Lopez-Royuela N Krzywinska E Garaude J Allende-Vega N. From tumor cell metabolism to tumor immune escape. Int J Biochem Cell Biol (2013) 45:106–13. 10.1016/j.biocel.2012.04.024 22568930

[170] Mocellin S Bronte V Nitti D. Nitric oxide, a double edged sword in cancer biology: searching for therapeutic opportunities. Med Res Rev (2007) 27:317–52. 10.1002/med.20092 16991100

[171] Cederbaum SD Yu H Grody WW Kern RM Yoo P Iyer RK. Arginases I and II: do their functions overlap? Mol Genet Metab (2004) 81(Suppl 1):S38–44. 10.1016/j.ymgme.2003.10.012 15050972

[172] Brito C Naviliat M Tiscornia AC Vuillier F Gualco G Dighiero G Peroxynitrite inhibits T lymphocyte activation and proliferation by promoting impairment of tyrosine phosphorylation and peroxynitrite-driven apoptotic death. J Immunol (1999) 162:3356–66. 10092790

[173] Aulak KS Miyagi M Yan L West KA Massillon D Crabb JW Proteomic method identifies proteins nitrated in vivo during inflammatory challenge. Proc Natl Acad Sci U S A (2001) 98:12056–61. 10.1073/pnas.221269198 11593016 PMC59826

[174] Nagaraj S Gupta K Pisarev V Kinarsky L Sherman S Kang L Altered recognition of antigen is a mechanism of CD8+ T cell tolerance in cancer. Nat Med (2007) 13:828–35. 10.1038/nm1609 17603493 PMC2135607

[175] Kasic T Colombo P Soldani C Wang CM Miranda E Roncalli M Modulation of human T-cell functions by reactive nitrogen species. Eur J Immunol (2011) 41:1843–9. 10.1002/eji.201040868 21480210

[176] Bronte V Kasic T Gri G Gallana K Borsellino G Marigo I Boosting antitumor responses of T lymphocytes infiltrating human prostate cancers. J Exp Med (2005) 201:1257–68. 10.1084/jem.20042028 15824085 PMC2213151

[177] Predonzani A Cali B Agnellini AH Molon B. Spotlights on immunological effects of reactive nitrogen species: when inflammation says nitric oxide. World J Exp Med (2015) 5:64–76. 10.5493/wjem.v5.i2.64 25992321 PMC4436941

[178] Rodriguez PC Quiceno DG Zabaleta J Ortiz B Zea AH Piazuelo MB Arginase I production in the tumor microenvironment by mature myeloid cells inhibits T-cell receptor expression and antigen-specific T-cell responses. Cancer Res (2004) 64:5839–49. 10.1158/0008-5472.CAN-04-0465 15313928

[179] Bronte V Zanovello P. Regulation of immune responses by L-arginine metabolism. Nat Rev Immunol (2005) 5:641–54. 10.1038/nri1668 16056256

[180] Dillon BJ Prieto VG Curley SA Ensor CM Holtsberg FW Bomalaski JS Incidence and distribution of argininosuccinate synthetase deficiency in human cancers: a method for identifying cancers sensitive to arginine deprivation. Cancer (2004) 100:826–33. 10.1002/cncr.20057 14770441

[181] Phillips MM Sheaff MT Szlosarek PW. Targeting arginine-dependent cancers with arginine-degrading enzymes: opportunities and challenges. Cancer Res Treat (2013) 45:251–62. 10.4143/crt.2013.45.4.251 24453997 PMC3893322

[182] Srivastava MK Sinha P Clements VK Rodriguez P Ostrand-Rosenberg S. Myeloid-derived suppressor cells inhibit T-cell activation by depleting cystine and cysteine. Cancer Res (2010) 70:68–77. 10.1158/0008-5472.CAN-09-2587 20028852 PMC2805057

[183] Munn DH Mellor AL. Indoleamine 2,3 dioxygenase and metabolic control of immune responses. Trends Immunol (2013) 34:137–43. 10.1016/j.it.2012.10.001 23103127 PMC3594632

[184] Vacchelli E Aranda F Eggermont A Sautes-Fridman C Tartour E Kennedy EP Trial watch: IDO inhibitors in cancer therapy. Oncoim-munology (2014) 3:e957994. 10.4161/21624011.2014.957994 25941578 PMC4292223

[185] Weinlich G Murr C Richardsen L Winkler C Fuchs D. Decreased serum tryptophan concentration predicts poor prognosis in malignant melanoma patients. Dermatology (2007) 214:8–14. 10.1159/000096906 17191041

[186] Godin-Ethier J Hanafi LA Piccirillo CA Lapointe R. Indoleamine 2,3-dioxygenase expression in human cancers: clinical and immunologic perspectives. Clin Cancer Res (2011) 17:6985–91. 10.1158/1078-0432.CCR-11-1331 22068654

[187] Gottfried E Kreutz M Mackensen A. Tumor metabolism as modulator of immune response and tumor progression. Semin Cancer Biol (2012) 22:335–41. 10.1016/j.semcancer.2012.02.009 22414910

[188] Mellor AL Chandler P Baban B Hansen AM Marshall B Pihkala J Specific subsets of murine dendritic cells acquire potent T cell regulatory functions following CTLA4-mediated induction of indoleamine 2,3 dioxygenase. Int Immunol (2004) 16:1391–401. 10.1093/intimm/dxh140 15351783

[189] Maciolek JA Pasternak JA Wilson HL. Metabolism of activated T lymphocytes. Curr Opin Immunol (2014) 27:60–74. 10.1016/j.coi.2014.01.006 24556090

[190] Rabinowitz JD White E Autophagy and metabolism. Science (2010) 330:1344–8. 10.1126/science.1193497 21127245 PMC3010857

[191] Egan DF Shackelford DB Mihaylova MM Gelino S Kohnz RA Mair W Phosphorylation of ULK1 (hATG1) by AMP-activated protein kinase connects energy sensing to mitophagy. Science (2011) 331:456–61. 10.1126/science.1196371 21205641 PMC3030664

[192] Mason EF Rathmell JC. Cell metabolism: an essential link between cell growth and apoptosis. Biochim Biophys Acta (2011) 1813:645–54. 10.1016/j.bbamcr.2010.08.011 20816705 PMC3010257

[193] Shackelford DB Shaw RJ. The LKB1-AMPK pathway: metabolism and growth control in tumour suppression. Nat Rev Cancer (2009) 9:563–75. 10.1038/nrc2676 19629071 PMC2756045

[194] Son HJ Lee J Lee SY Kim EK Park MJ Kim KW Metformin attenuates experimental autoimmune arthritis through reciprocal regulation of Th17/Treg balance and osteoclastogenesis. Mediators Inflamm (2014) 2014:973986. 10.1155/2014/973986 25214721 PMC4158168

[195] Murray IA Patterson AD Perdew GH Aryl hydrocarbon receptor ligands in cancer: friend and foe. Nat Rev Cancer (2014) 14:801–14. 10.1038/nrc3846 25568920 PMC4401080

[196] Chen W Jin W Hardegen N Lei KJ Li L Marinos N Conversion of peripheral CD4+CD25- naive T cells to CD4+CD25+ regulatory T cells by TGF-beta induction of transcription factor Foxp3. J Exp Med (2003) 198:1875–86. 10.1084/jem.20030152 14676299 PMC2194145

[197] Curiel TJ Coukos G Zou L Alvarez X Cheng P Mottram P Specific recruitment of regulatory T cells in ovarian carcinoma fosters immune privilege and predicts reduced survival. Nat Med (2004) 10:942–9. 10.1038/nm1093 15322536

[198] Wilke CM Wu K Zhao E Wang G Zou W. Prognostic significance of regulatory T cells in tumor. Int J Cancer (2010) 127:748–58. 10.1002/ijc.25464 20473951

[199] Gerriets VA Kishton RJ Johnson MO Cohen S Siska PJ Nichols AG Foxp3 and toll-like receptor signaling balance Treg cell anabolic metabolism for suppression. Nat Immunol (2016) 17:1459–66. 10.1038/ni.3577 27695003 PMC5215903

[200] Macintyre AN Gerriets VA Nichols AG Michalek RD Rudolph MC Deoliveira D The glucose transporter Glut1 is selectively essential for CD4 T cell activation and effector function. Cell Metab (2014) 20:61–72. 10.1016/j.cmet.2014.05.004 24930970 PMC4079750

[201] Sinclair LV Rolf J Emslie E Shi YB Taylor PM Cantrell DA. Control of amino-acid transport by antigen receptors coordinates the metabolic reprogramming essential for T cell differentiation. Nat Immunol (2013) 14:500–8. 10.1038/ni.2556 23525088 PMC3672957

[202] Wieman HL Wofford JA Rathmell JC. Cytokine stimulation promotes glucose uptake via phosphatidylinositol-3 kinase/Akt regulation of Glut1 activity and trafficking. Mol Biol Cell (2007) 18:1437–46. 10.1091/mbc.E06-07-0593 17301289 PMC1838986

[203] McCracken AN Edinger AL. Nutrient transporters: the Achilles’ heel of anabolism. Trends Endocrinol Metab (2013) 24:200–8. 10.1016/j.tem.2013.01.002 23402769 PMC3617053

[204] Parry RV Chemnitz JM Frauwirth KA Lanfranco AR Braunstein I Kobayashi SV CTLA-4 and PD-1 receptors inhibit T-cell activation by distinct mechanisms. Mol Cell Biol (2005) 25:9543–53. 10.1128/MCB.25.21.9543-9553.2005 16227604 PMC1265804

[205] Frauwirth KA Riley JL Harris MH Parry RV Rathmell JC Plas DR The CD28 signaling pathway regulates glucose metabolism. Immunity (2002) 16:769–77. 10.1016/S1074-7613(02)00323-0 12121659

[206] Doering TA Crawford A Angelosanto JM Paley MA Ziegler CG Wherry EJ. Network analysis reveals centrally connected genes and pathways involved in CD8+ T cell exhaustion versus memory. Immunity (2012) 37:1130–44. 10.1016/j.immuni.2012.08.021 23159438 PMC3749234

[207] Shimatani K Nakashima Y Hattori M Hamazaki Y Minato N. PD-1+ memory phenotype CD4+ T cells expressing C/EBPalpha underlie T cell immunodepression in senescence and leukemia. Proc Natl Acad Sci U S A (2009) 106:15807–12. 10.1073/pnas.0908805106 19805226 PMC2739871

[208] Patsoukis N Li L Sari D Petkova V Boussiotis VA. PD-1 increases PTEN phosphatase activity while decreasing PTEN protein stability by inhibiting casein kinase 2. Mol Cell Biol (2013) 33:3091–8. 10.1128/MCB.00319-13 23732914 PMC3753920

[209] Saha A Aoyama K Taylor PA Koehn BH Veenstra RG Panoskaltsis-Mortari A Host programmed death ligand 1 is dominant over programmed death ligand 2 expression in regulating graft-versus-host disease lethality. Blood (2013) 122:3062–73. 10.1182/blood-2013-05-500801 24030385 PMC3811178

[210] Walker LS Sansom DM. Confusing signals: recent progress in CTLA-4 biology. Trends Immunol (2015) 36:63–70. 10.1016/j.it.2014.12.001 25582039 PMC4323153

[211] Chen X Fosco D Kline DE Meng L Nishi S Savage PA PD-1 regulates extrathymic regulatory T-cell differentiation. Eur J Immunol (2014) 44:2603–16. 10.1002/eji.201344423 24975127 PMC4165701

[212] Ha TY. The role of regulatory T cells in cancer. Immune Netw (2009) 9:209–35. 10.4110/in.2009.9.6.209 20157609 PMC2816955

[213] Whiteside TL. What are regulatory T cells (Treg) regulating in cancer and why? Semin Cancer Biol (2012) 22:327–34. 10.1016/j.semcancer.2012.03.004 22465232 PMC3385925

[214] Patsoukis N Bardhan K Chatterjee P Sari D Liu B Bell LN PD-1 alters T-cell metabolic reprogramming by inhibiting glycolysis and promoting lipolysis and fatty acid oxidation. Nat Commun (2015) 6:6692. 10.1038/ncomms7692 25809635 PMC4389235

[215] Barsoum IB Koti M Siemens DR Graham CH. Mechanisms of hypoxia-mediated immune escape in cancer. Cancer Res (2014) 74:7185–90. 10.1158/0008-5472.CAN-14-2598 25344227

[216] Arreygue-Garcia NA Daneri-Navarro A del Toro-Arreola A Cid-Arregui A Gonzalez-Ramella O Jave-Suarez LF Augmented serum level of major histocompatibility complex class I-related chain A (MICA) protein and reduced NKG2D expression on NK and T cells in patients with cervical cancer and precursor lesions. BMC Cancer (2008) 8:16 10.1186/1471-2407-8-16 18208618 PMC2270854

[217] Barsoum IB Hamilton TK Li X Cotechini T Miles EA Siemens DR Hypoxia induces escape from innate immunity in cancer cells via increased expression of ADAM10: role of nitric oxide. Cancer Res (2011) 71:7433–41. 10.1158/0008-5472.CAN-11-2104 22006996

[218] Ahmadzadeh M Johnson LA Heemskerk B Wunderlich JR Dudley ME White DE Tumor antigen-specific CD8 T cells infiltrating the tumor express high levels of PD-1 and are functionally impaired. Blood (2009) 114:1537–44. 10.1182/blood-2008-12-195792 19423728 PMC2927090

[219] Page DB Postow MA Callahan MK Allison JP Wolchok JD. Immune modulation in cancer with antibodies. Annu Rev Med (2014) 65:185–202. 10.1146/annurev-med-092012-112807 24188664

[220] Chen TC Wu CT Wang CP Hsu WL Yang TL Lou PJ Associations among pretreatment tumor necrosis and the expression of HIF-1alpha and PD-L1 in advanced oral squamous cell carcinoma and the prognostic impact thereof. Oral Oncol (2015) 51:1004–10. 10.1016/j.oraloncology.2015.08.011 26365985

[221] Barsoum IB Smallwood CA Siemens DR Graham CH. A mechanism of hypoxia-mediated escape from adaptive immunity in cancer cells. Cancer Res (2014) 74:665–74. 10.1158/0008-5472.CAN-14-2598 24336068

[222] Guido C Whitaker-Menezes D Capparelli C Balliet R Lin Z Pestell RG Metabolic reprogramming of cancer-associated fibroblasts by TGF-beta drives tumor growth: connecting TGF-beta signaling with “Warburg-like” cancer metabolism and L-lactate production. Cell Cycle (2012) 11:3019–35. 10.4161/cc.21384 22874531 PMC3442913

[223] Guido C Whitaker-Menezes D Lin Z Pestell RG Howell A Zimmers TA Mitochondrial fission induces glycolytic reprogramming in cancer-associated myofibroblasts, driving stromal lactate production, and early tumor growth. Oncotarget (2012) 3:798–810. 10.18632/oncotarget.574 22878233 PMC3478457

[224] Cai M He J Xiong J Tay LW Wang Z Rog C Phospholipase D1-regulated autophagy supplies free fatty acids to counter nutrient stress in cancer cells. Cell Death Dis (2016) 7:e2448. 10.1038/cddis.2016.355 27809301 PMC5260880

[225] Schafer M Werner S. Cancer as an overhealing wound: an old hypothesis revisited. Nat Rev Mol Cell Biol (2008) 9:628–38. 10.1038/nrm2455 18628784

[226] Pavlides S Whitaker-Menezes D Castello-Cros R Flomenberg N Witkiewicz AK Frank PG The reverse Warburg effect: aerobic glycolysis in cancer associated fibroblasts and the tumor stroma. Cell Cycle (2009) 8:3984–4001. 10.4161/cc.8.23.10238 19923890

[227] Martinez-Outschoorn UE Balliet R Lin Z Whitaker-Menezes D Birbe RC Bombonati A BRCA1 mutations drive oxidative stress and glycolysis in the tumor microenvironment: implications for breast cancer prevention with antioxidant therapies. Cell Cycle (2012) 11:4402–13. 10.4161/cc.22776 23172369 PMC3552923

[228] Xu XD Shao SX Cao YW Yang XC Shi HQ Wang YL The study of energy metabolism in bladder cancer cells in co-culture conditions using a microfluidic chip. Int J Clin Exp Med (2015) 8:12327–36. 26550142 PMC4612827

[229] Yu T Yang G Hou Y Tang X Wu C Wu XA Cytoplasmic GPER translocation in cancer-associated fibroblasts mediates cAMP/PKA/CREB/glycolytic axis to confer tumor cells with multidrug resistance. Oncogene (2016) 1–15. 10.1038/onc.2016.370 27721408

[230] Arcucci A Ruocco MR Granato G Sacco AM Montagnani S. Cancer: an oxidative crosstalk between solid tumor cells and cancer associated fibroblasts. Biomed Res Int (2016) 2016:4502846. 10.1155/2016/4502846 27595103 PMC4993917

[231] Pertega-Gomes N Vizcaino JR Attig J Jurmeister S Lopes C Baltazar F. A lactate shuttle system between tumour and stromal cells is associated with poor prognosis in prostate cancer. BMC Cancer (2014) 14:352. 10.1186/1471-2407-14-352 24886074 PMC4039335

[232] Kim Y Choi JW Lee JH Kim YS Expression of lactate/H(+) symporters MCT1 and MCT4 and their chaperone CD147 predicts tumor progression in clear cell renal cell carcinoma: immunohistochemical and the Cancer Genome Atlas data analyses. Hum Pathol (2015) 46:104–12. 10.1016/j.humpath.2014.09.013 25456395

[233] Miranda-Goncalves V Granja S Martinho O Honavar M Pojo M Costa BM Hypoxia-mediated upregulation of MCT1 expression supports the glycolytic phenotype of glioblastomas. Oncotarget (2016) 7:46335–53. 10.18632/oncotarget.10114 27331625 PMC5216802

[234] Knudsen ES Balaji U Freinkman E McCue P Witkiewicz AK. Unique metabolic features of pancreatic cancer stroma: relevance to the tumor compartment, prognosis, and invasive potential. Oncotarget (2016) 7(48):78396–411. 10.18632/oncotarget.11893 27623078 PMC5346648

[235] Burbridge MF West DC Atassi G Tucker GC. The effect of extracellular pH on angiogenesis in vitro. Angiogenesis (1999) 3:281–8. 10.1023/A:1009092511894 14517427

[236] Dong L Li Z Leffler NR Asch AS Chi JT Yang LV. Acidosis activation of the proton-sensing GPR4 receptor stimulates vascular endothelial cell inflammatory responses revealed by transcriptome analysis. PLoS One (2013) 8:e61991. 10.1371/journal.pone.0061991 23613998 PMC3628782

[237] Zhang D Wang Y Shi Z Liu J Sun P Hou X Metabolic reprogramming of cancer-associated fibroblasts by IDH3alpha downregulation. Cell Rep (2015) 10:1335–48. 10.1016/j.celrep.2015.02.006 25732824

[238] Ko YH Domingo-Vidal M Roche M Lin Z Whitaker-Menezes D Seifert E TP53-inducible glycolysis and apoptosis regulator (TIGAR) metabolically reprograms carcinoma and stromal cells in breast cancer. J Biol Chem (2016) 291:26291–303. 10.1074/jbc.M116.740209 27803158 PMC5159492

[239] Shim H Dolde C Lewis BC Wu CS Dang G Jungmann RA c-Myc transactivation of LDH-A: implications for tumor metabolism and growth. Proc Natl Acad Sci U S A (1997) 94:6658–63. 10.1073/pnas.94.13.6658 9192621 PMC21214

[240] Matoba S Kang JG Patino WD Wragg A Boehm M Gavrilova O p53 regulates mitochondrial respiration. Science (2006) 312:1650–3. 10.1126/science.1126863 16728594

[241] Won KY Lim SJ Kim GY Kim YW Han SA Song JY Regulatory role of p53 in cancer metabolism via SCO2 and TIGAR in human breast cancer. Hum Pathol (2012) 43:221–8. 10.1016/j.humpath.2011.04.021 21820150

[242] Choi J Stradmann-Bellinghausen B Yakubov E Savaskan NE Regnier-Vigouroux A. Glioblastoma cells induce differential glutamatergic gene expressions in human tumor-associated microglia/macrophages and monocyte-derived macrophages. Cancer Biol Ther (2015) 16:1205–13. 10.1080/15384047.2015.1056406 26047211 PMC4623498

[243] Seo JW Choi J Lee SY Sung S Yoo HJ Kang MJ Autophagy is required for PDAC glutamine metabolism. Sci Rep (2016) 6:37594. 10.1038/srep37594 27892481 PMC5124864

[244] Abramczyk H Surmacki J Kopec M Olejnik AK Lubecka-Pietruszewska K Fabianowska-Majewska K. The role of lipid droplets and adipocytes in cancer. Raman imaging of cell cultures: MCF10A, MCF7, and MDA-MB-231 compared to adipocytes in cancerous human breast tissue. Analyst (2015) 140:2224–35. 10.1039/c4an01875c 25730442

[245] Nieman KM Kenny HA Penicka CV Ladanyi A Buell-Gutbrod R Zillhardt MR Adipocytes promote ovarian cancer metastasis and provide energy for rapid tumor growth. Nat Med (2011) 17:1498–503. 10.1038/nm.2492 22037646 PMC4157349

[246] Carter JC Church FC. Mature breast adipocytes promote breast cancer cell motility. Exp Mol Pathol (2012) 92:312–7. 10.1016/j.yexmp.2012.03.005 22445926

[247] D’Esposito V Passaretti F Hammarstedt A Liguoro D Terracciano D Molea G Adipocyte-released insulin-like growth factor-1 is regulated by glucose and fatty acids and controls breast cancer cell growth in vitro. Diabetologia (2012) 55:2811–22. 10.1007/s00125-012-2629-7 22798065 PMC3433668

[248] Balaban S Shearer RF Lee LS van Geldermalsen M Schreuder M Shtein HC Adipocyte lipolysis links obesity to breast cancer growth: adipocyte-derived fatty acids drive breast cancer cell proliferation and migration. Cancer Metab (2017) 5:1. 10.1186/s40170-016-0163-7 28101337 PMC5237166

[249] Wen YA Xing X Harris JW Zaytseva YY Mitov MI Napier DL Adipocytes activate mitochondrial fatty acid oxidation and autophagy to promote tumor growth in colon cancer. Cell Death Dis (2017) 8:e2593. 10.1038/cddis.2017.21 28151470 PMC5386470

[250] Guaita-Esteruelas S Guma J Masana L Borras J. The peritumoural adipose tissue microenvironment and cancer. The roles of fatty acid binding protein 4 and fatty acid binding protein 5. Mol Cell Endocrinol (2017). 10.1016/j.mce.2017.02.002 28163102

[251] Gazi E Gardner P Lockyer NP Hart CA Brown MD Clarke NW. Direct evidence of lipid translocation between adipocytes and prostate cancer cells with imaging FTIR microspectroscopy. J Lipid Res (2007) 48:1846–56. 10.1194/jlr.M700131-JLR200 17496269

[252] Pascual G Avgustinova A Mejetta S Martin M Castellanos A Attolini CS Targeting metastasis-initiating cells through the fatty acid receptor CD36. Nature (2017) 541:41–5. 10.1038/nature20791 27974793

[253] Li Z Kang Y. Lipid metabolism fuels cancer’s spread. Cell Metab (2017) 25:228–30. 10.1016/j.cmet.2017.01.016 28178563

[254] Vander Heiden MG Cantley LC Thompson CB. Understanding the Warburg effect: the metabolic requirements of cell proliferation. Science (2009) 324:1029–33. 10.1126/science.1160809 19460998 PMC2849637

[255] Liu Y Zuckier LS Ghesani NV. Dominant uptake of fatty acid over glucose by prostate cells: a potential new diagnostic and therapeutic approach. Anticancer Res (2010) 30:369–74. 20332441

[256] Deep G Schlaepfer IR Aberrant lipid metabolism promotes prostate cancer: role in cell survival under hypoxia and extracellular vesicles biogenesis. Int J Mol Sci (2016) 17:1061–74. 10.3390/ijms17071061 PMC4964437 27384557

[257] Staron MM Gray SM Marshall HD Parish IA Chen JH Perry CJ The transcription factor FoxO1 sustains expression of the inhibitory receptor PD-1 and survival of antiviral CD8(+) T cells during chronic infection. Immunity (2014) 41:802–14. 10.1016/j.immuni.2014.10.013 25464856 PMC4270830

[258] Farooque A Singh N Adhikari JS Afrin F Dwarakanath BS. Enhanced antitumor immunity contributes to the radio-sensitization of Ehrlich ascites tumor by the glycolytic inhibitor 2-deoxy-D-glucose in mice. PLoS One (2014) 9:e108131. 10.1371/journal.pone.0108131 25248151 PMC4172770

[259] Farooque A Afrin F Adhikari JS Dwarakanath BS. Polarization of macrophages towards M1 phenotype by a combination of 2-deoxy-d-glucose and radiation: implications for tumor therapy. Immunobiology (2016) 221:269–81. 10.1016/j.imbio.2015.10.009 26597503

[260] Hubert S Rissiek B Klages K Huehn J Sparwasser T Haag F Extracellular NAD+ shapes the Foxp3+ regulatory T cell compartment through the ART2-P2X7 pathway. J Exp Med (2010) 207:2561–8. 10.1084/jem.20091154 20975043 PMC2989765

[261] Leone RD Lo YC Powell JD. A2aR antagonists: next generation checkpoint blockade for cancer immunotherapy. Comput Struct Biotechnol J (2015) 13:265–72. 10.1016/j.csbj.2015.03.008 25941561 PMC4415113

[262] Sonveaux P Vegran F Schroeder T Wergin MC Verrax J Rabbani ZN Targeting lactate-fueled respiration selectively kills hypoxic tumor cells in mice. J Clin Invest (2008) 118:3930–42. 10.1172/JCI36843 19033663 PMC2582933

[263] Blagosklonny MV. Flavopiridol, an inhibitor of transcription: implications, problems and solutions. Cell Cycle (2004) 3:1537–42. 10.4161/cc.3.12.1278 15539947

[264] Lee K Zhang H Qian DZ Rey S Liu JO Semenza GL. Acriflavine inhibits HIF-1 dimerization, tumor growth, and vascularization. Proc Natl Acad Sci U S A (2009) 106:17910–5. 10.1073/pnas.0909353106 19805192 PMC2764905

[265] Zhang H Qian DZ Tan YS Lee K Gao P Ren YR Digoxin and other cardiac glycosides inhibit HIF-1alpha synthesis and block tumor growth. Proc Natl Acad Sci U S A (2008) 105:19579–86. 10.1073/pnas.0809763105 19020076 PMC2604945

[266] Yang QC Zeng BF Shi ZM Dong Y Jiang ZM Huang J Inhibition of hypoxia-induced angiogenesis by trichostatin A via suppression of HIF-1a activity in human osteosarcoma. J Exp Clin Cancer Res (2006) 25:593–9. 17310851

[267] Doedens AL Phan AT Stradner MH Fujimoto JK Nguyen JV Yang E Hypoxia-inducible factors enhance the effector responses of CD8(+) T cells to persistent antigen. Nat Immunol (2013) 14:1173–82. 10.1038/ni.2714 24076634 PMC3977965

[268] Carmeliet P Jain RK. Principles and mechanisms of vessel normalization for cancer and other angiogenic diseases. Nat Rev Drug Discov (2011) 10:417–27. 10.1038/nrd3455 21629292

[269] Maione F Capano S Regano D Zentilin L Giacca M Casanovas O Semaphorin 3A overcomes cancer hypoxia and metastatic dissemination induced by antiangiogenic treatment in mice. J Clin Invest (2012) 122:1832–48. 10.1172/JCI58976 22484816 PMC3336974

[270] Sukumar M Liu J Ji Y Subramanian M Crompton JG Yu Z Inhibiting glycolytic metabolism enhances CD8+ T cell memory and antitumor function. J Clin Invest (2013) 123:4479–88. 10.1172/JCI69589 24091329 PMC3784544

[271] Pearce EL Walsh MC Cejas PJ Harms GM Shen H Wang LS Enhancing CD8 T-cell memory by modulating fatty acid metabolism. Nature (2009) 460:103–7. 10.1038/nature08097 19494812 PMC2803086

[272] Ben Sahra I Regazzetti C Robert G Laurent K Le Marchand-Brustel Y Auberger P Metformin, independent of AMPK, induces mTOR inhibition and cell-cycle arrest through REDD1. Cancer Res (2011) 71:4366–72. 10.1158/0008-5472.CAN-10-1769 21540236

[273] Blandino G Valerio M Cioce M Mori F Casadei L Pulito C Metformin elicits anticancer effects through the sequential modulation of DICER and c-MYC. Nat Commun (2012) 3:865. 10.1038/ncomms1859 22643892

[274] Waickman AT Powell JD. mTOR, metabolism, and the regulation of T-cell differentiation and function. Immunol Rev (2012) 249:43–58. 10.1111/j.1600-065X.2012.01152.x 22889214 PMC3419491

[275] Rao RR Li Q Odunsi K Shrikant PA. The mTOR kinase determines effector versus memory CD8+ T cell fate by regulating the expression of transcription factors T-bet and Eomesodermin. Immunity (2010) 32:67–78. 10.1016/j.immuni.2009.10.010 20060330 PMC5836496

[276] Li C Capan E Zhao Y Zhao J Stolz D Watkins SC Autophagy is induced in CD4+ T cells and important for the growth factor-withdrawal cell death. J Immunol (2006) 177:5163–8. 10.4049/jimmunol.177.8.5163 17015701

[277] Law BK. Rapamycin: an anti-cancer immunosuppressant? Crit Rev Oncol Hematol (2005) 56:47–60. 10.1016/j.critrevonc.2004.09.009 16039868

[278] Sharma MD Shinde R McGaha TL Huang L Holmgaard RB Wolchok JD The PTEN pathway in Tregs is a critical driver of the suppressive tumor microenvironment. Sci Adv (2015) 1:e1500845. 10.1126/sciadv.1500845 26601142 PMC4640592

[279] Yang S de Souza P Alemao E Purvis J. Quality of life in patients with advanced renal cell carcinoma treated with temsirolimus or interferon-alpha. Br J Cancer (2010) 102:1456–60. 10.1038/sj.bjc.6605647 20461090 PMC2869160

[280] Motzer RJ Escudier B Oudard S Hutson TE Porta C Bracarda S Efficacy of everolimus in advanced renal cell carcinoma: a double-blind, randomised, placebo-controlled phase III trial. Lancet (2008) 372:449–56. 10.1016/S0140-6736(08)61039-9 18653228

[281] Martelli AM Chiarini F Evangelisti C Cappellini A Buontempo F Bressanin D Two hits are better than one: targeting both phosphatidylinositol 3-kinase and mammalian target of rapamycin as a therapeutic strategy for acute leukemia treatment. Oncotarget (2012) 3:371–94. 10.18632/oncotarget.477 22564882 PMC3380573

[282] Wang H Wang L Zhang Y Wang J Deng Y Lin D. Inhibition of glycolytic enzyme hexokinase II (HK2) suppresses lung tumor growth. Cancer Cell Int (2016) 16:9. 10.1186/s12935-016-0280-y 26884725 PMC4755025

[283] Botzer LE Maman S Sagi-Assif O Meshel T Nevo I Yron I Hexokinase 2 is a determinant of neuroblastoma metastasis. Br J Cancer (2016) 114:759–66. 10.1038/bjc.2016.26 26986252 PMC4984856

[284] Brown J Effects of 2-deoxyglucose on carbohydrate metablism: review of the literature and studies in the rat. Metabolism (1962) 11:1098–112. 13873661

[285] McComb RB Yushok WD Metabolism of ascites tumor cells. Iv. Enzymatic reactions involved in adenosinetriphosphate degradation induced by 2-deoxyglucose. Cancer Res (1964) 24:198–205. 14115684

[286] Dwarakanath BS. Cytotoxicity, radiosensitization, and chemosensitization of tumor cells by 2-deoxy-D-glucose in vitro. J Cancer Res Ther (2009) 5(Suppl 1):S27–31. 10.4103/0973-1482.55137 20009290

[287] Mohanti BK Rath GK Anantha N Kannan V Das BS Chandramouli BA Improving cancer radiotherapy with 2-deoxy-D-glucose: phase I/II clinical trials on human cerebral gliomas. Int J Radiat Oncol Biol Phys (1996) 35:103–11. 10.1016/S0360-3016(96)85017-6 8641905

[288] Singh D Banerji AK Dwarakanath BS Tripathi RP Gupta JP Mathew TL Optimizing cancer radiotherapy with 2-deoxy-d-glucose dose escalation studies in patients with glioblastoma multiforme. Strahlenther Onkol (2005) 181:507–14. 10.1007/s00066-005-1320-z 16044218

[289] Dwarakanath BS Singh D Banerji AK Sarin R Venkataramana NK Jalali R Clinical studies for improving radiotherapy with 2-deoxy-D-glucose: present status and future prospects. J Cancer Res Ther (2009) 5(Suppl 1):S21–6. 10.4103/0973-1482.55136 20009289

[290] Venkataramanaa NK Venkatesh PK Dwarakanath BS Vani S. Protective effect on normal brain tissue during a combinational therapy of 2-deoxy-d-glucose and hypofractionated irradiation in malignant gliomas. Asian J Neurosurg (2013) 8:9–14. 10.4103/1793-5482.110274 23741257 PMC3667464

[291] Marko AJ Miller RA Kelman A Frauwirth KA. Induction of glucose metabolism in stimulated T lymphocytes is regulated by mitogen-activated protein kinase signaling. PLoS One (2010) 5:e15425. 10.1371/journal.pone.0015425 21085672 PMC2978105

[292] Strum SB Adalsteinsson O Black RR Segal D Peress NL Waldenfels J Case report: sodium dichloroacetate (DCA) inhibition of the “Warburg Effect” in a human cancer patient: complete response in non-Hodgkin’s lymphoma after disease progression with rituximab-CHOP. J Bioenerg Biomembr (2013) 45:307–15. 10.1007/s10863-013-9516-x 23263938

[293] Jiang P Du W Wu M. Regulation of the pentose phosphate pathway in cancer. Protein Cell (2014) 5:592–602. 10.1007/s13238-014-0082-8 25015087 PMC4112277

[294] Tsouko E Khan AS White MA Han JJ Shi Y Merchant FA Regulation of the pentose phosphate pathway by an androgen receptor-mTOR-mediated mechanism and its role in prostate cancer cell growth. Oncogenesis (2014) 3:e103. 10.1038/oncsis.2014.18 24861463 PMC4035695

[295] Sharma PK Dwarakanath BS Varshney R. Radiosensitization by 2-deoxy-D-glucose and 6-aminonicotinamide involves activation of redox sensitive ASK1-JNK/p38MAPK signaling in head and neck cancer cells. Free Radic Biol Med (2012) 53:1500–13. 10.1016/j.freeradbiomed.2012.07.001 22824861

[296] Varshney R Dwarakanath B Jain V. Radiosensitization by 6-aminonicotinamide and 2-deoxy-D-glucose in human cancer cells. Int J Radiat Biol (2005) 81:397–408. 10.1080/09553000500148590 16076755

[297] Varshney R Gupta S Dwarakanath BS. Radiosensitization of murine Ehrlich ascites tumor by a combination of 2-deoxy-D-glucose and 6-aminonicotinamide. Technol Cancer Res Treat (2004) 3:659–63. 10.1177/153303460400300616 15560724

[298] Zhu W Ye L Zhang J Yu P Wang H Ye Z PFK15, a small molecule inhibitor of PFKFB3, induces cell cycle arrest, apoptosis and inhibits invasion in gastric cancer. PLoS One (2016) 11:e0163768. 10.1371/journal.pone.0163768 27669567 PMC5036843

[299] Li S Wu L Feng J Li J Liu T Zhang R In vitro and in vivo study of epigallocatechin-3-gallate-induced apoptosis in aerobic glycolytic hepatocellular carcinoma cells involving inhibition of phosphofructokinase activity. Sci Rep (2016) 6:28479. 10.1038/srep28479 27349173 PMC4923908

[300] Lian N Jin H Zhang F Wu L Shao J Lu Y Curcumin inhibits aerobic glycolysis in hepatic stellate cells associated with activation of adenosine monophosphate-activated protein kinase. IUBMB Life (2016) 68:589–96. 10.1002/iub.1518 27278959

[301] Cantelmo AR Conradi LC Brajic A Goveia J Kalucka J Pircher A Inhibition of the glycolytic activator PFKFB3 in endothelium induces tumor vessel normalization, impairs metastasis, and improves chemotherapy. Cancer Cell (2016) 30:968–85. 10.1016/j.ccell.2016.10.006 27866851 PMC5675554

[302] Majkowska-Skrobek G Augustyniak D Lis P Bartkowiak A Gonchar M Ko YH Killing multiple myeloma cells with the small molecule 3-bromopyruvate: implications for therapy. Anticancer Drugs (2014) 25:673–82. 10.1097/CAD.0000000000000094 24557015

[303] Lis P Dylag M Niedzwiecka K Ko YH Pedersen PL Goffeau A The HK2 dependent “Warburg effect” and mitochondrial oxidative phosphorylation in cancer: targets for effective therapy with 3-bromopyruvate. Molecules (2016) 21:E1730 10.3390/molecules21121730 27983708 PMC6273842

[304] Doherty JR Cleveland JL. Targeting lactate metabolism for cancer therapeutics. J Clin Invest (2013) 123:3685–92. 10.1172/JCI69741 23999443 PMC3754272

[305] Papaldo P Lopez M Cortesi E Cammilluzzi E Antimi M Terzoli E Addition of either lonidamine or granulocyte colony-stimulating factor does not improve survival in early breast cancer patients treated with high-dose epirubicin and cyclophosphamide. J Clin Oncol (2003) 21:3462–8. 10.1200/JCO.2003.03.034 12972521

[306] Di Cosimo S Ferretti G Papaldo P Carlini P Fabi A Cognetti F. Lonidamine: efficacy and safety in clinical trials for the treatment of solid tumors. Drugs Today (Barc) (2003) 39:157–74. 10.1358/dot.2003.39.3.799451 12730701

[307] Fang J Quinones QJ Holman TL Morowitz MJ Wang Q Zhao H The H+-linked monocarboxylate transporter (MCT1/SLC16A1): a potential therapeutic target for high-risk neuroblastoma. Mol Pharmacol (2006) 70:2108–15. 10.1124/mol.106.026245 17000864

[308] Yu H Zhang H Dong M Wu Z Shen Z Xie Y Metabolic reprogramming and AMPKalpha1 pathway activation by caulerpin in colorectal cancer cells. Int J Oncol (2017) 50:161–72. 10.3892/ijo.2016.3794 27922662

[309] Molon B Ugel S Del Pozzo F Soldani C Zilio S Avella D Chemokine nitration prevents intratumoral infiltration of antigen-specific T cells. J Exp Med (2011) 208:1949–62. 10.1084/jem.20101956 21930770 PMC3182051

[310] Muller AJ DuHadaway JB Donover PS Sutanto-Ward E Prendergast GC. Inhibition of indoleamine 2,3-dioxygenase, an immunoregulatory target of the cancer suppression gene Bin1, potentiates cancer chemotherapy. Nat Med (2005) 11:312–9. 10.1038/nm1196 15711557

[311] Liu X Shin N Koblish HK Yang G Wang Q Wang K Selective inhibition of IDO1 effectively regulates mediators of antitumor immunity. Blood (2010) 115:3520–30. 10.1182/blood-2009-09-246124 20197554

[312] Balachandran VP Cavnar MJ Zeng S Bamboat ZM Ocuin LM Obaid H Imatinib potentiates antitumor T cell responses in gastrointestinal stromal tumor through the inhibition of Ido. Nat Med (2011) 17:1094–100. 10.1038/nm.2438 21873989 PMC3278279

[313] Dietz AB Souan L Knutson GJ Bulur PA Litzow MR Vuk-Pavlovic S. Imatinib mesylate inhibits T-cell proliferation in vitro and delayed-type hypersensitivity in vivo. Blood (2004) 104:1094–9. 10.1182/blood-2003-12-4266 15100154

[314] Nika K Soldani C Salek M Paster W Gray A Etzensperger R Constitutively active Lck kinase in T cells drives antigen receptor signal transduction. Immunity (2010) 32:766–77. 10.1016/j.immuni.2010.05.011 20541955 PMC2996607

[315] Luo J Hong Y Tao X Wei X Zhang L Li Q An indispensable role of CPT-1a to survive cancer cells during energy stress through rewiring cancer metabolism. Tumour Biol (2016) 37:15795–804. 10.1007/s13277-016-5382-6 27739027

[316] Qian J Chen Y Meng T Ma L Meng L Wang X Molecular regulation of apoptotic machinery and lipid metabolism by mTORC1/mTORC2 dual inhibitors in preclinical models of HER2+/PIK3CAmut breast cancer. Oncotarget (2016) 7(41):67071–86. 10.18632/oncotarget.11490 27563814 PMC5341858

[317] Zeng H Yang K Cloer C Neale G Vogel P Chi H. mTORC1 couples immune signals and metabolic programming to establish T(reg)-cell function. Nature (2013) 499:485–90. 10.1038/nature12297 23812589 PMC3759242

[318] Tontonoz P Hu E Spiegelman BM. Stimulation of adipogenesis in fibroblasts by PPAR gamma 2, a lipid-activated transcription factor. Cell (1994) 79:1147–56. 10.1016/0092-8674(94)90006-X 8001151

[319] Demetri GD Fletcher CD Mueller E Sarraf P Naujoks R Campbell N Induction of solid tumor differentiation by the peroxisome proliferator-activated receptor-gamma ligand troglitazone in patients with liposarcoma. Proc Natl Acad Sci U S A (1999) 96:3951–6. 10.1073/pnas.96.7.3951 10097144 PMC22401

[320] Tebbe C Chhina J Dar SA Sarigiannis K Giri S Munkarah AR Metformin limits the adipocyte tumor-promoting effect on ovarian cancer. Oncotarget (2014) 5:4746–64. 10.18632/oncotarget.2012 24970804 PMC4148096

[321] Martinez-Outschoorn UE Balliet RM Rivadeneira DB Chiavarina B Pavlides S Wang C Oxidative stress in cancer associated fibroblasts drives tumor-stroma co-evolution: a new paradigm for understanding tumor metabolism, the field effect and genomic instability in cancer cells. Cell Cycle (2010) 9:3256–76. 10.4161/cc.9.16.12553 20814239 PMC3041164

[322] Ibrahim-Hashim A Wojtkowiak JW de Lourdes Coelho Ribeiro M Estrella V Bailey KM Cornnell HH Free base lysine increases survival and reduces metastasis in prostate cancer model. J Cancer Sci Ther (2011). 10.4172/1948-5956.S1-004 24032073 PMC3768133

[323] Ibrahim Hashim A Cornnell HH Coelho Ribeiro Mde L Abrahams D Cunningham J Lloyd M Reduction of metastasis using a non-volatile buffer. Clin Exp Metastasis (2011) 28:841–9. 10.1007/s10585-011-9415-7 21861189 PMC3213349

[324] Ibrahim-Hashim A Cornnell HH Abrahams D Lloyd M Bui M Gillies RJ Systemic buffers inhibit carcinogenesis in TRAMP mice. J Urol (2012) 188:624–31. 10.1016/j.juro.2012.03.113 22704445 PMC3694604

[325] Reichert M Steinbach JP Supra P Weller M. Modulation of growth and radiochemosensitivity of human malignant glioma cells by acidosis. Cancer (2002) 95:1113–9. 10.1002/cncr.10767 12209698

[326] Gerweck LE Vijayappa S Kozin S. Tumor pH controls the in vivo efficacy of weak acid and base chemotherapeutics. Mol Cancer Ther (2006) 5:1275–9. 10.1158/1535-7163.MCT-06-0024 16731760

[327] Morimura T Fujita K Akita M Nagashima M Satomi A. The proton pump inhibitor inhibits cell growth and induces apoptosis in human hepatoblastoma. Pediatr Surg Int (2008) 24:1087–94. 10.1007/s00383-008-2229-2 18712525

[328] von Schwarzenberg K Wiedmann RM Oak P Schulz S Zischka H Wanner G Mode of cell death induction by pharmacological vacuolar H+-ATPase (V-ATPase) inhibition. J Biol Chem (2013) 288:1385–96. 10.1074/jbc.M112.412007 23168408 PMC3543021

[329] Kastelein F Spaander MC Steyerberg EW Biermann K Valkhoff VE Kuipers EJ Proton pump inhibitors reduce the risk of neoplastic progression in patients with Barrett’s esophagus. Clin Gastroenterol Hepatol (2013) 11:382–8. 10.1016/j.cgh.2012.11.014 23200977

[330] Ihnatko R Kubes M Takacova M Sedlakova O Sedlak J Pastorek J Extracellular acidosis elevates carbonic anhydrase IX in human glioblastoma cells via transcriptional modulation that does not depend on hypoxia. Int J Oncol (2006) 29:1025–33. 10.3892/ijo.29.4.1025 16964400

[331] Supuran CT Development of small molecule carbonic anhydrase IX inhibitors. BJU Int (2008) 101(Suppl 4):39–40. 10.1111/j.1464-410X.2008.07648.x 18430122

[332] Lou Y McDonald PC Oloumi A Chia S Ostlund C Ahmadi A Targeting tumor hypoxia: suppression of breast tumor growth and metastasis by novel carbonic anhydrase IX inhibitors. Cancer Res (2011) 71:3364–76. 10.1158/0008-5472.CAN-10-4261 21415165

[333] Justus CR Dong L Yang LV. Acidic tumor microenvironment and pH-sensing G protein-coupled receptors. Front Physiol (2013) 4:354. 10.3389/fphys.2013.00354 24367336 PMC3851830

[334] Yang L Achreja A Yeung TL Mangala LS Jiang D Han C Targeting stromal glutamine synthetase in tumors disrupts tumor microenvironment-regulated cancer cell growth. Cell Metab (2016) 24:685–700. 10.1016/j.cmet.2016.10.011 27829138 PMC7329194

[335] Singh S Pandey S Bhatt AN Chaudhary R Bhuria V Kalra N Chronic dietary administration of the glycolytic inhibitor 2-Deoxy-D-Glucose (2-DG) inhibits the growth of implanted Ehrlich’s ascites tumor in mice. PLoS One (2015) 10:e0132089. 10.1371/journal.pone.0132089 26135741 PMC4489743

[336] Newton R Priyadharshini B Turka LA. Immunometabolism of regulatory T cells. Nat Immunol (2016) 17:618–25. 10.1038/ni.3466 27196520 PMC5006394

[337] Kelly B O’Neill LA. Metabolic reprogramming in macrophages and dendritic cells in innate immunity. Cell Res (2015) 25:771–84. 10.1038/cr.2015.68 26045163 PMC4493277

[338] Pandey S Singh S Anang V Bhatt AN Natarajan K Dwarakanath BS. Pattern recognition receptors in cancer progression and metastasis. Cancer Growth Metastasis (2015) 8:25–34. 10.4137/CGM.S24314 26279628 PMC4514171

